# RNA-Seq effectively monitors gene expression in *Eutrema salsugineum* plants growing in an extreme natural habitat and in controlled growth cabinet conditions

**DOI:** 10.1186/1471-2164-14-578

**Published:** 2013-08-28

**Authors:** Marc J Champigny, Wilson WL Sung, Vasile Catana, Rupa Salwan, Peter S Summers, Susan A Dudley, Nicholas J Provart, Robin K Cameron, G Brian Golding, Elizabeth A Weretilnyk

**Affiliations:** 1 Department of Biology, McMaster University, Hamilton, Ontario L8S 4K1, Canada; 2 Department of Cell and Systems Biology, University of Toronto, Toronto, Ontario M5S 3B2, Canada

**Keywords:** *Eutrema salsugineum*, *Thellungiella salsuginea*, Transcriptome profiling, RNA-Seq, Salt tolerance, Natural plant populations, Single nucleotide polymorphisms, Phenotypic plasticity, Ecological genomics, Natural field conditions, Halophyte, Extremophile, Plant stress tolerance traits

## Abstract

**Background:**

The investigation of extremophile plant species growing in their natural environment offers certain advantages, chiefly that plants adapted to severe habitats have a repertoire of stress tolerance genes that are regulated to maximize plant performance under physiologically challenging conditions. Accordingly, transcriptome sequencing offers a powerful approach to address questions concerning the influence of natural habitat on the physiology of an organism. We used RNA sequencing of *Eutrema salsugineum,* an extremophile relative of *Arabidopsis thaliana*, to investigate the extent to which genetic variation and controlled versus natural environments contribute to differences between transcript profiles*.*

**Results:**

Using 10 million cDNA reads, we compared transcriptomes from two natural *Eutrema* accessions (originating from Yukon Territory, Canada and Shandong Province, China) grown under controlled conditions in cabinets and those from Yukon plants collected at a Yukon field site. We assessed the genetic heterogeneity between individuals using single-nucleotide polymorphisms (SNPs) and the expression patterns of 27,016 genes. Over 39,000 SNPs distinguish the Yukon from the Shandong accessions but only 4,475 SNPs differentiated transcriptomes of Yukon field plants from an inbred Yukon line. We found 2,989 genes that were differentially expressed between the three sample groups and multivariate statistical analyses showed that transcriptomes of individual plants from a Yukon field site were as reproducible as those from inbred plants grown under controlled conditions. Predicted functions based upon gene ontology classifications show that the transcriptomes of field plants were enriched by the differential expression of light- and stress-related genes, an observation consistent with the habitat where the plants were found.

**Conclusion:**

Our expectation that comparative RNA-Seq analysis of transcriptomes from plants originating in natural habitats would be confounded by uncontrolled genetic and environmental factors was not borne out. Moreover, the transcriptome data shows little genetic variation between laboratory Yukon *Eutrema* plants and those found at a field site. Transcriptomes were reproducible and biological associations meaningful whether plants were grown in cabinets or found in the field. Thus RNA-Seq is a valuable approach to study native plants in natural environments and this technology can be exploited to discover new gene targets for improved crop performance under adverse conditions.

## Background

Determining how wild plants have adapted to extreme environments can provide useful insight into how all plants respond to stress. In particular, researchers measure genetic differences among populations found in different environments [1,2]. A classic approach is to compare trait differences among populations when they are grown in the same environment as in a common garden. Comparative genomics studies using natural accessions can also provide insights into traits associated with the adaptation of an organism to a given habitat [3]. The genetic basis underlying traits of interest can be studied using the natural genetic variation between accessions combined with standard genetic approaches such as marker based mapping strategies to identify trait-associated allele(s). However, traits expressed in an environment are the consequence of both genetic and environmental influences [4], so that an adaptation displayed under natural conditions may not be detectable in a controlled environment chamber [5].

*Eutrema salsugineum* (Pall.) Al-Shehbaz & Warwick (also known as *Thellungiella salsuginea* (Pall.) O.E. Schulz) offers an ideal system to explore hypotheses about plant adaptations to extreme environments. This native crucifer is closely related to the genetic model plant *Arabidopsis thaliana* but displays a far greater capacity to withstand freezing temperatures, water deficits, and saline soils [6-9]. Two naturally occurring accessions have been the subject of several studies with one accession originating from the Yukon Territory, Canada and the second from Shandong Province, China. These accessions have evolved under contrasting natural habitats with the one in Canada growing under a semi-arid, subarctic climate and the accession in China under a temperate climate subject to more frequent precipitation.

Yukon *Eutrema* plants show plasticity to field versus controlled environment chambers with respect to morphology, stress-responsive genes and metabolites [7]. Plants in the field show prominent cauline leaves and, for the most part, lack rosette leaves while plants in cabinets have many rosette but few cauline leaves. At the level of the metabolome, salt-stressed plants in growth chambers accumulate proline in a osmo-responsive manner [7,9,10], but plants in a saline field site had a much lower proline content that was comparable to chamber-grown plants that had not been salt stressed. Manipulation of salt and nitrogen treatments under controlled growth conditions demonstrated that proline accumulated with high nitrogen content in saline culture media. Plants exposed to salt when on low nitrogen accumulated carbohydrates as did plants found under natural conditions [7]. Thus comparisons involving field and chamber-grown plants can provide novel and valuable insights into the breadth of traits subject to phenotypic plasticity and how they may contribute towards the adaptation of plants to their natural environment.

A limited set of comparisons among transcript profiles of field and chamber-grown Yukon *Eutrema* plants was reported by Guevara et al. [7] using a microarray chip representing 3,628 unigenes. They found statistically significant, biologically meaningful gene expression patterns in comparisons between plants stressed under controlled conditions in cabinets and field plants. However, the information that microarray data can provide is limited [11,12]. One issue is poor genome coverage; the chip used surveys a comparatively small sampling of the predicted coding capacity for the full *Eutrema* genome [13,14]. Another problem is that microarray data are relative and require incorporation of a control comparison, which may not exist for a field sample. Microarrays are also relatively insensitive to distinguishing between genes with multiple family members [12] and this could be further complicated by an indeterminate level of genetic and environmental variation among plants from a natural population in their native habitat. In this investigation we assessed the suitability of an alternative approach to comparative transcriptome profiling based upon next generation sequencing (NGS). We used high throughput mRNA sequencing technology (RNA-Seq) that exploits the sensitivity and genome-wide resolving power of NGS platforms. This approach offers a fully quantitative measure of mRNA abundance that is particularly suitable for studying organisms where little is known about their transcriptome and a full genome chip is not commercially available [11].

Ultimately, the challenge of applying RNA-Seq technology to plants found in the field is its sensitivity. RNA-Seq generates large datasets and interpretable patterns from field plants may be difficult to discern given that direct comparisons with plants grown under controlled environment conditions cannot be made. In this study we included natural accessions of *Eutrema* from Shandong and the Yukon and we compared inbred cabinet-grown Yukon and Shandong plants and Yukon plants sampled in the field. We hypothesized that 1) sequences would differ more between the two accessions, while Yukon individuals sampled in the field or raised in cabinets would show far fewer differences. This hypothesis was confirmed by an analysis of single-nucleotide polymorphisms (SNPs) using transcriptomes of the Yukon plants (field and cabinet-grown) and Shandong plants (cabinet-grown). We hypothesized that 2) the gene expression results would differ between Yukon plants from the field site and growth cabinets, which represent radically different environments. While this prediction held true, the transcriptomes from Yukon and Shandong plants grown at the same time in the same growth cabinet were also very different emphasizing the important interaction between genotype and environment. We hypothesized that 3) transcriptomes from individual plants selected at random at a Yukon field site would be more variable compared to those of individuals selected from the same single-seed descent line and grown together under controlled conditions in growth cabinets. However, this expectation was not borne out. The pattern of gene expression for individual plants of a common genotype that originated from the same environment were similar regardless of where the plants had grown together, showing transcriptomes to be consistent between individual plants of a sample group. While one might anticipate the high sensitivity afforded by NGS to yield transcriptome data that is more difficult to interpret when genetic and environmental variation are uncontrolled variables, our study does not support this hypothesis. Instead, we found RNA-Seq to be a viable approach to compare gene expression patterns of *Eutrema* growing either in controlled environment chambers or *in situ* in the complex and changeable environment of the Yukon.

## Results and discussion

### Plant selection and experimental design

Nine plants consisting of three individuals from each of three experimental groups were profiled by RNA-Seq. Transcriptomes derived from these plants were expected to display heterogeneity arising from differences between environmental exposure and genotype.

One group consisted of three plants selected in late June of 2005 from a field site approximately 40 km NW of Whitehorse in the Yukon Territory of Canada. Details concerning the soil composition, meteorological conditions and morphology of plants growing at this field site were reported by Guevara et al. [7]. *Eutrema* plants at this field site have an upright habit with rosette leaves being either very small or absent. For analysis, cauline leaves from randomly selected, individual plants of undetermined age were harvested from the field for transcriptome profiling and these samples are referred to as YF (Yukon Field) 1, YF2, and YF3.

A second group of three plants of the Yukon accession were grown in a controlled environment chamber under daylength conditions set to resemble a Yukon summer day (see Methods). *Eutrema* grown under these laboratory conditions have a prominent rosette [7]. Rosette leaves of three four week-old plants produced in growth cabinets were selected for transcriptome profiling and these samples are referred to as YC (Yukon Cabinet) 1, YC2, and YC3. Seeds from plants growing at the same Yukon field site were pooled and maintained as a bulked collection from which some seeds were used to establish inbred lines. Two of the plants in this group, YC1 and YC2, were products of single-seed descent from the same parent for five generations while the third, YC3, was grown from a randomly selected seed from the bulked pool.

The third group profiled was composed of three *Eutrema* plants of the Shandong accession. Plants were subjected to single-seed descent for four generations to increase their genetic homogeneity. Sibling Shandong *Eutrema* plants from a single-seed descent line were grown in the same cabinet and hence under identical conditions as the YC1 and YC2 samples. Rosette leaves of three four week-old Shandong chamber-grown plants were harvested for transcriptome profiling and these samples are referred to as SC (Shandong Cabinet) 1, SC2, and SC3.

The field plants (YF) and YC3 are not products of single-seed descent lines and hence were expected to show greater genetic heterogeneity than the inbred YC1 and YC2 plants. In terms of comparative genetic variability we expected that the greatest heterogeneity would be found between Shandong and Yukon plants. Thus use of the Shandong genotype provided a source of genetic variation and had the additional benefit in facilitating transcriptome assembly through access to its fully sequenced genome as a reference [13,14].

### RNA-Seq of *Eutrema* samples

Massively parallel sequencing of nine cDNA libraries generated from *Eutrema* leaf samples of individual plants was conducted on the Roche 454 GS FLX platform. When we began our transcriptome sequencing, a genome assembly for *E. salsugineum* was not available making the longer read lengths afforded by 454 technology advantageous for *de novo* transcriptome assembly [15,16]. The draft genome releases of the Shandong accession of *Eutrema* have since aided our transcriptome assembly, allowing unambiguous association of individual sequence reads with *Eutrema* gene loci. Our RNA-Seq methodology therefore exemplifies a “mapping-first” or genome-guided transcriptomics approach [17].

The draft genome of the Shandong accession of *Eutrema* released by the Joint Genome Institute (JGI - *Thellungiella halophila* Genome Project 2011: http://www.phytozome.net/thellungiella.php), shows the genome to be 243.1 Mb in size, distributed among 639 scaffolds with preliminary annotation available for 26,351 protein-coding loci [14]. The Beijing Genomics Institute (BGI) has also released a genome assembly of Shandong *E. salsugineum,* which is 234 Mb in size, distributed among 2,682 scaffolds. The predicted number of protein-coding genes in this assembly is 28,457 [13]. In this report the comparisons or citations to gene annotations, relative positions, and sequences are made with reference to the JGI draft genome release given its earlier availability as a public resource. We have since compared our mapped reads to both genome sequences and found negligible differences in the alignments produced. For example, of the total number of reads obtained from nine cDNA libraries, only 0.3% were aligned to the BGI genome release and not the JGI release.

Information concerning cDNA library sequencing and read alignments to the JGI draft genome is presented in Table 1. Adaptor sequences and regions of poor quality were removed from the raw reads resulting in a total of 9,758,824 high quality reads. The mean read length across all libraries was 282 bp, and the frequency distribution of read lengths obtained was similar in all sequencing runs (not shown).

**Table 1:** **Metrics for reads, alignments and levels of rRNA in *Eutrema***

**Library**	**Reads**					
	**Before trimming**	**After trimming**	**Length (mean ± SD)**	**Alignment to JGI genome**		**rRNA**
				**Unique**	**Non-unique**	
	***n***	***N***	***bp***	***%***	***%***	***%***
SC1	1,505,998	1,410,766	315 ± 115	97.4	1.7	0.63
SC2	654,380	594,219	289 ± 111	95.3	3.1	2.0
SC3	733,305	648,435	268 ± 109	93.9	2.7	0.85
YC1	1,322,008	1,233,425	288 ± 105	94.8	2.7	0.90
YC2	726,603	685,135	312 ± 113	96.1	1.7	0.53
YC3	1,529,004	1,372,930	227 ± 84	95.4	2.9	1.4
YF1	1,501,857	1,415,995	292 ± 103	95.1	2.7	1.7
YF2	1,411,966	1,309,781	318 ± 111	94.8	3.0	2.2
YF3	1,349,259	1,088,138	236 ± 100	92.3	3.6	0.94
All 9 libraries	10,734,380	9,758,824	282 ± 110	95.1	2.7	1.3

Non-unique reads map to more than one locus in the JGI Genome. The last column indicates the proportion of trimmed reads derived from the expression of rRNA genes in each library.

Trimmed reads derived from each library were aligned against the genome release using open-source GMAP software. GMAP is a splice-aware aligner that allows mapping of cDNA sequences against a reference containing introns and gaps [18]. Alignments were categorized into three groups consisting of uniquely mapped reads, non-uniquely mapped reads, and unmapped reads that could not be aligned to any locus in the reference and, in view of this difficulty, were not considered further in our analyses (Table 1). The vast majority of cDNA sequences in each library (92.3 - 97.4%) aligned to a single location in the reference but a small number (1.7 - 3.6%) mapped to multiple locations. We predicted that many of the non-uniquely aligned cDNA reads were expression products of genes with members sharing extensive sequence identity. Interestingly, a large fraction of these reads (109,624/263,015 reads or 42% across all libraries) mapped to a single region on scaffold 14 of the JGI release. A custom annotation for this region (Additional file 1: Figure S1) shows that it includes a tandem array of five duplicated ribosomal RNA gene clusters with 5.8S, 18S, and 25S rRNA genes present in each cluster. The array of rRNA genes is a conserved genomic feature that is also present in a similar arrangement on chromosome 2 of *Arabidopsis*[19]. This array of rRNA was useful in estimating the level of rRNA transcripts contributing to our cDNA libraries prepared from polyadenylated mRNAs. On average, only 1.27% of the reads in each library were derived from rRNA (Table 1), indicating that a vast majority of sequences were derived from mRNAs.

Genomic studies of geographically disparate accessions of *Arabidopsis thaliana* have identified thousands of small genome-wide differences as well as more major genome rearrangements (http://www.1001genomes.org) [20]. For example, a fraction of the Col-0 reference genome is absent in the genomes of other accessions [21,22]. In our study, cDNA sequences were aligned to the draft genome assembly of the Shandong accession. Alignments of the Shandong-derived sequence reads (from SC1, SC2, and SC3) were well matched to the reference genome and only a small fraction (0.8-3.4%) of the total reads remained unmatched following alignment (Table 1). We expected that reads derived from the Yukon accession would find less frequent agreement with the draft genome due to polymorphisms between the accessions. However, using this genome-guided RNA-Seq approach, the libraries derived from cabinet-grown and the naturally occurring Yukon *Eutrema* performed as well as the Shandong libraries with, on average, only 2.4% of the total reads from these sources remaining unmatched (Table 1). This outcome is consistent with a high degree of similarity with respect to gene composition and sequence identity between the genomes of the two accessions.

### Expression of previously unannotated genes supported by Yukon *Eutrema* transcripts

A total of 26,351 *E. salsugineum* protein-coding loci were annotated by JGI using transcript evidence from 1.6 million 454 cDNA reads (http://www.phytozome.net/thellungiella.php). We reasoned that our *Eutrema* libraries comprising over 10 million reads would contain transcripts derived from non-abundantly expressed genes or environment-specific genes that have not yet been annotated. We used Cufflinks software in a reference-based transcript assembly approach [23] and found read support that enriched the existing complement of annotated genes by 665 loci. Between JGI and this study, there is transcript support for 27,016 loci, a number that compares well with 28,457 predicted genes in the BGI Shandong *E. salsugineum* genome release [13] and 28,901 protein-coding genes for the closely related *Eutrema parvulum* (also known as *T. parvula*) [24]. A summary of these newly annotated genes is presented in Additional file 2. A BLAST analysis of the 665 genes indicated that 344 (52%) have a match to an *Arabidopsis thaliana* gene and using Plant RefSeq and the *E. parvulum* databases we matched 34 more genes (http://ftp.ncbi.nlm.nih.gov/refseq/release/plant) [24]. This analysis left 287 (43%) genes of the 665 genes as unidentified based upon available information. Of the 665 newly annotated genes, expression associated with 101 was only detected in the Yukon-genotype transcriptomes of which 15 genes were only expressed in plants growing at the Yukon field site. Thus our RNA-Seq analysis of the Yukon accession that included plants found in the natural environment provided evidence for the expression of a number of genes in regions of the *E. salsugineum* genome for which transcript support was previously unavailable.

### Sequence polymorphisms in *Eutrema* accessions

We evaluated the extent of genetic diversity between the two accessions of *Eutrema* and among the Yukon laboratory and field plants. For this analysis we used a bioinformatics approach to identify SNPs and small insertions/deletions (InDels) in the RNA-Seq datafiles of our plant samples compared to the JGI reference genome sequence.

The mpileup function of SAMtools [25] was used to detect sequence differences within reads that aligned to a unique location in the JGI reference. At each candidate position, GATK [26] software identified SNPs and InDels for each library. This initial set of 92,814 SNPs and 15,060 InDels was then subjected to filters to eliminate the number of differences that might be attributed to sequencing errors. Custom Perl scripts eliminated SNPs supported by fewer than five sequence reads across nine libraries and InDels with an mpileup quality score less than 999. Polymorphisms detected at a frequency supported by less than 1% of informative reads were also excluded from further analysis. The resulting set of high quality polymorphisms comprises 74,550 two-allele SNPs (Additional file 3) and 1,429 two-allele InDels (Additional file 4). We anticipate that future *E. salsugineum* genome releases will position the current suite of genes onto improved physical maps. With this prospect in mind, we have provided 100 bp of genomic sequence flanking each polymorphism listed so that they can be more easily located within the present as well as future *E. salsugineum* genome releases (Additional file 3 and Additional file 4). While these polymorphisms were supported by the sequences of multiple reads, to ensure their accuracy we also subjected a group to testing by q-PCR and high resolution melting (HRM) analysis (Table 2). Of 40 loci containing accession-specific polymorphisms (33 SNPs and 7 InDels), HRM only failed to detect one polymorphism in an amplicon associated with a Class IV SNP (T → A, see Table 2). Efforts to sequence this locus using traditional Sanger sequencing also failed so the status of this single SNP was unresolved for technical reasons. Thus our computational strategy identified SNP and InDel polymorphisms accurately enabling us to use the 74,550 SNPs to evaluate both the distribution of polymorphisms among genes and the genetic diversity between the *Eutrema* plants used in this study.

**Table 2:** **A subset of *Eutrema *polymorphisms that differentiate the two accessions**

**Position of polymorphism**	**JGI*Eutrema*locus identifier**	**Base change(s) Yukon: Shandong**	**Coverage Yukon: Shandong**
Scaffold 1: 4,440,114	Thhalv10025531m	A:G	90:40
Scaffold 2: 6,032,225	Thhalv10014327m	A:G	167:93
Scaffold 2: 8,916,704	Thhalv10013555m	A:G	1180:335
Scaffold 3: 5,423,444	Thhalv10028955m	A:G	48:25
Scaffold 6: 2,15 9,339	Thhalv10003756m	A:G	48:26
Scaffold 6: 5,412,324	Thhalv10005526m	A:G	61:44
Scaffold 13: 7,815,985	Thhalv10021402m	A:G	134:89
Scaffold 13: 6,172,584	Thhalv10020197m	A:G	768:79
Scaffold 13: 5,369,287	Thhalv10020602m	A:G	72:50
Scaffold 16: 1,662,648	Thhalv10010065m	A:G	48:31
Scaffold 16: 1,475,722	Thhalv10010527m	A:G	70:31
Scaffold 19: 2,648,494	Thhalv10005990m	A:G	74:39
Scaffold 19: 944,324	Thhalv10005892m	A:G	1990:647
Scaffold 1: 7,216,530	Thhalv10024615m	T:A	67:107
Scaffold 10: 6, 554,603	Thhalv10016361m	T:A	100:51
Scaffold 13: 8,863,896	Thhalv10020900m	T:A	181:24
Scaffold 14: 4 221,543	Thhalv10027724m	T:A	62:52
Scaffold 15: 544,264	Thhalv10000015m	T:A	60:43
Scaffold 22: 987,812	Thhalv10001310m	T:A	63:46
Scaffold 5: 1,539,747	Thhalv10007601m	T:A	44:31
Scaffold 8: 1,169,845	Thhalv10023594m	T:A	53:21
Scaffold 1: 6,103,206	Thhalv10026365m	T:A	254:130
Scaffold 10: 8,782,193	Thhalv10017224m	T:A	58:34
Scaffold 10: 11,862,224	Thhalv10017066m	T:A	101:65
Scaffold 13: 3,668,845	Thhalv10021205m	T:A	94:97
Scaffold 16: 1, 960,564	not annotated	T:A	167:22
Scaffold 2: 4,381,656	Thhalv10013572m	T:A	135:125
Scaffold 2: 4,455,202	Thhalv10012443m	T:A	1006:198
Scaffold 2: 9,054,220	Thhalv10013543m	T:A	74:23
Scaffold 5: 6,470,821	Thhalv10009562m	T:A	22:82
Scaffold 5: 9,933,756	Thhalv10007762m	T:A	248:532
Scaffold 5: 11,358,486	Thhalv10008740m	T:A	51:48
Scaffold 1: 3,499,240 *	Thhalv10024883m	T:A	118:39
Scaffold 19: 1,451,367	Thhalv10006024m	TAA: -	6:23
Scaffold 20: 897,166	Thhalv10000834m	- :A	14:3
Scaffold 10: 2,975,499	Thhalv10016162m	- :TGAGTCTAG	41:35
Scaffold 13: 5,622,589	Thhalv10021362m	AA: -	32:22
Scaffold 16: 4,729,391	Thhalv10010831m	- :CAGTA	216:45
Scaffold 7: 1,061,288	Thhalv10011242m	- :GTGAATCTG	172:116
Scaffold 9: 674,593	not annotated	- :T	133:89

High Resolution Melting (HRM) was conducted on genomic DNA purified from single, cabinet-grown Yukon and Shandong plants. Position of the polymorphism on the JGI scaffold is indicated as well as its association with a gene locus in the JGI *Eutrema* genome release. Only one computationally predicted SNP (indicated by *) could not be confirmed by HRM. Coverage refers to the number of uniquely mapped sequencing reads aligning to a polymorphism-associated locus in each accession.

The ability to locate SNPs within individual genes allowed us to identify genes that contain a high number or density of SNPs. Of the 74,550 SNPs described, 9,087 fall within currently unannotated regions of the genome and the remainder (65,463 SNPs) were found within the 26,351 *Eutrema* genes annotated by JGI and the additional 665 genes annotated in this study (Additional file 2), yielding an average of 2.4 SNPs per gene across all of our transcriptomes. The 100 annotated loci exhibiting SNP densities in excess of 25 SNPs/kb are presented in Additional file 5. Loci with anomalously high SNP-density could be generated as artifacts through problems in genome assembly and/or improper read alignment of closely related genes to a scaffold position. However, the SNP-dense loci on this list are likely genuine given that they are supported by multiple reads and, as discussed below, they encode gene products similar to those identified as highly polymorphic in other studies.

The *Eutrema* orthologue of *SCAR2* (Thhalv10002989m.g) has a SNP density of 95.2 SNPs/kb and it is the locus with the greatest density of SNPs for which a function of the gene can be surmised. *SCAR2* is a gene whose product is implicated in regulating cytoskeletal activities involved in morphogenesis [27]. The locus with the highest total number of SNPs has 237 SNPs distributed among 32 exons (Thhalv10012578m.g - 75 SNPs/kb) and this gene encodes a putative calcium-transporting ATPase. In *A. lyrata*, polymorphism-dense genes encode calcium and magnesium transporters and metal-tolerance proteins that are implicated in this species’ ability to grow in soils featuring a low calcium-to-magnesium ratio termed serpentine [28]. The soil at the Yukon field site where *E. salsugineum* grows is serpentine [7], suggesting that SNP-rich genes like Thhalv10012578m.g should be evaluated for a role in the local adaptation of Yukon *Eutrema* to its natural habitat.

Genes exhibiting high levels of diversity are hypothesized by population theory to contain balanced polymorphisms that can indicate adaptive variation [29]. In a study of high-diversity genes differing between the Col-0 and L*er* accessions of *Arabidopsis*, Cork and Purugganan [30] described 29 loci of particularly high variation residing within three genomic regions. However, none of the 100 most SNP-rich *Eutrema* genes (Additional file 5) are orthologous to these *Arabidopsis* genes. To gain further insight into the possible biological functions represented by the 100 most SNP-dense genes, this list was subjected to a Gene Ontology (GO) analysis to identify categories enriched among these genes compared to the predicted coding capacity of the 27,016 annotated genes (Additional file 6) in the *Eutrema* genome. In this analysis only two categories were over-represented, namely “defense response” (*p* = 4.14 × 10^-9^) and “response to biotic or abiotic stress” (*p* = 1.75 × 10^-7^). A BLAST-based homology analysis against the Plant Resistance Gene Database identified 32 of the 100 most SNP-dense loci as putative *R* genes (http://prgdb.crg.eu/wiki/Main_Page) [31]. This is consistent with results of a recent genomic study of multiple *Arabidopsis* accessions noting that *R* genes were the group of genes exhibiting the greatest level of genetic variability across accessions [32] and *R* genes are known to be subject to local adaptation [33,34].

### Assessment of genetic diversity among *Eutrema* plants

We used the 74,550 SNPs to estimate the extent of genetic diversity among the *Eutrema* plants profiled. To increase the confidence of these comparisons we added another filter requiring that a minimum of two reads support a polymorphism between groups. Our rationale is that no decision regarding a SNP should be supported by a single read from one group of plants due to concerns regarding sequencing errors [35,36]. The number of SNPs differing between the JGI reference sequence and the SC, YC, and YF groups in all pair-wise comparisons is presented in Table 3. We expected and found the fewest SNPs between plants of similar genotype. There were 2,839 SNPs between the JGI genome sequence and the SC transcriptomes that we profiled and of these, 101 SNPs were unique to this comparison. Similarly, only 4,475 SNPs were found following a comparison between YC and YF transcriptomes. To provide a context for evaluating whether these types of comparisons represent a significant number of differences, we can compare our results to those of Ossowski et al. [37] who re-sequenced the Col-0 *Arabidopsis* line used in their laboratory and identified 1,172 SNP differences with respect to the TIGR version 5 Col-0 genome release. The authors highlighted that a significant fraction (82%) of the Col-0 specific SNPs discovered in their genome re-sequencing study could be reasonably attributed to errors in the reference sequence [37].

**Table 3:** **Genetic diversity between *Eutrema *groups**

**Groups compared**	**Total SNPs in comparison**	**SNPs unique to comparison**
JGI, SC	2,839	101
JGI, YC	52,732	3,289
JGI, YF	52,512	2,483
SC, YC	41,160	n/a
SC, YF	39,367	n/a
YC, YF	4,475	n/a

74,550 two-allele SNPs differentiate the plant groups and the JGI reference genome from each other. Some SNPs were also unique to only one two-way comparison. All SNPs were identified as variants of the JGI reference so n/a denotes that SNPs unique to comparisons between transcriptomes could not be identified. For inclusion, SNPs detected between groups of transcriptomes had to be supported by more than two SNPs per plant group.

We identified a large number of SNPs that distinguish the two accessions from each other, with the number found varying between 39,367 and 52,732 in different comparisons (JGI genome or SC versus YC or YF; Table 3). A total of 16,454 were accession-specific SNPs in that they are associated with only one allele in all four Shandong plants (JGI reference, SC1, SC2, SC3) and only one alternative allele in all six Yukon plants profiled (Additional file 3). There were also 240 InDels that delineated the Shandong accession from the Yukon accession (Additional file 4). In a related context, Gan et al. [32] calculated the number of SNPs supported by RNA-Seq reads that differed between the TAIR 10 Col-0 reference and their genome assemblies for 18 *Arabidopsis* accessions as ranging from 57,604 SNPs in Can-0 to 118,965 SNPs in Po-0. Thus the number of SNPs we identified as differing between the two *Eutrema* accessions in this RNA-Seq study falls within the number of SNPs identified as distinguishing various *Arabidopsis* accessions from Col-0.

Transcriptome profiling of multiple individuals by a reference-guided RNA-Seq approach provides adequate sequencing depth within coding regions to identify potential errors within the reference genome. The 101 Shandong-specific polymorphisms mentioned above could be genuine differences between closely related lines but they are also candidate loci that may represent errors in the JGI reference. We made use of the BGI Shandong *E. salsugineum*[13] reference genome and determined that, for 45% of the SNPs differing between the JGI genome release and the SC transcriptomes, the BGI reference sequence was identical to that of our SC transcriptomes. The remaining 55% of SNP-associated loci located in the BGI genome were in agreement with the JGI genome release [14]. Given that these SNP-associated loci were supported by multiple sequence reads in each of three Shandong plants profiled, these Shandong-specific SNPs merit closer scrutiny as possible errors within the current genome releases.

The Shandong plants and two of the cabinet-grown Yukon plants (YC1 and YC2) were products of lines subject to a single-seed descent procedure to increase their genetic similarity. The number of SNPs (2,839) distinguishing the SC plants from the JGI reference is consistent with an expectation of high genome sequence similarity (Table 3). In contrast, the plants at the Yukon field site were not subjected to deliberate homogenization strategies so we were surprised to find only 4,475 SNPs differing between the transcriptome sequences of cabinet-grown *Eutrema* (YC) and YF plants found growing in a natural Yukon habitat (Table 3). We have no reason to believe that three randomly selected plants from the field site in 2005 and plants we self-pollinated to create inbred lines in the laboratory beginning in 2002 share the same pedigree. However, factors conducive for the development of a reproductively isolated and genetically homogenous natural population include the self-fertile nature of this plant and the patchy distribution of salt flats in the Yukon.

We used the frequency of SNP heterozygosity within individual plants as a means to quantify the extent of genetic variation between the three groups of natural and cabinet-grown *Eutrema* plants. By this criterion, SNP heterozygosity ranged from 1.5% to 2.6% within plants in the SC group, 5.4% to 7.5% in the YC group and 5.6% to 7.0% in the YF group (Table 4). We believe that these are overestimates of heterozygosity because this metric assumes that each SNP is inherited independently. Nonetheless these results are similar to estimates of heterozygosity in populations of self-fertile *Arabidopsis thaliana* that average approximately 3% but can range from 1% to as high as 5% in some stands [38]. Of particular note, the heterozygosity of the YC3 individual that was deliberately not subjected to single-seed descent was 7.3% and thus comparable to estimates for the Yukon single-seed descent line (YC1, YC2) and the natural field plants (YF) (Table 4). The higher homozygosity within the Shandong plants (SC) may reflect a lower rate of outcrossing in the Shandong accession in comparison to the Yukon. However, the more likely explanation is that the Shandong *Eutrema* seeds we received were already inbred and we subjected them to multiple generations of single seed descent.

**Table 4:** Heterozygosity of individual plants

**Plant**	**Heterozygosity (%)**
SC1	2.6
SC2	1.5
SC3	2.5
YC1	7.5
YC2	5.4
YC3	7.3
YF1	7.0
YF2	8.1
YF3	5.6

For each plant, the percentage of SNPs present as heterozygous alleles compared to the total number of SNPs was calculated. YF1, YF2, and YF3 plants were randomly selected from the field site; YC3 was derived from a bulked pool of seeds collected at the field site; SC1, SC2, SC3, YC1, and YC2 were derived from single seed descent procedures.

**Figure 1:**
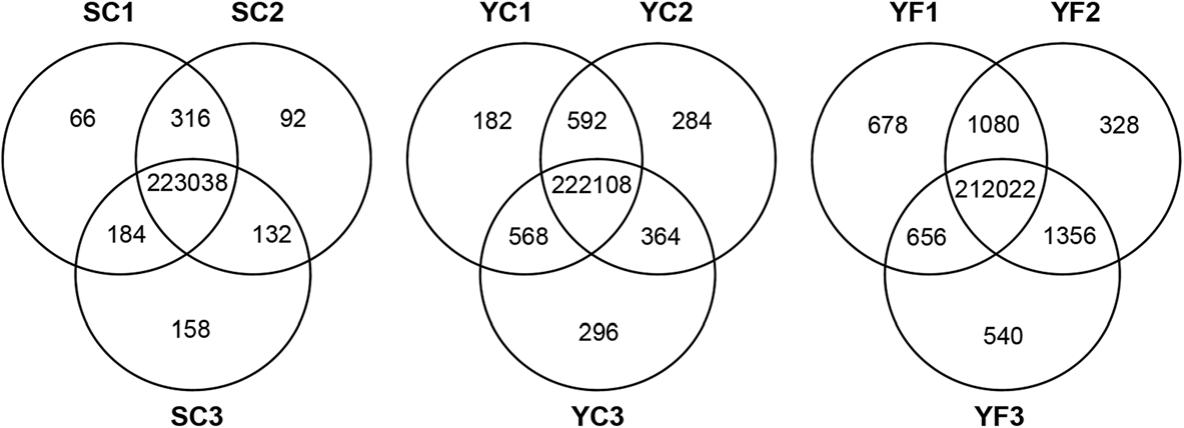
**Genetic diversity within *Eutrema *accessions.** Two-allele SNPs identified in this study were discriminated at a resolution of individual plants. Genetic diversity within groups was investigated by recording each SNP-associated allele as either homozygous or heterozygous in each plant. The number of SNP-associated alleles that were shared between plants of a particular group are indicated in the overlapping sectors of the Venn diagrams.

The results shown in Table 3 provide an estimate of polymorphic differences between groups and support the conclusion that within-accession genetic variation is low. However, these comparisons reflect the pooled contributions of SNPs found in three individuals, making it possible that a single plant made a disproportionate contribution towards the estimated genetic heterogeneity. For example, while only 4,475 SNPs were identified between YC and YF transcriptomes, it is possible that the majority of those SNPs were due to differences in a single YF plant and this consideration would lead to a different conclusion about the genetic uniformity of the field plants. To assess the genetic diversity among the individuals within the three groups, we used a subset of SNP-associated loci from the 74,550 SNPs for which sequence coverage exceeded five reads in each of the three individual plants within a group. In this analysis we determined whether a given SNP-associated allele was shared (or not) by other plants within the group. The results of this analysis are shown in the Venn diagrams of Figure 1. The region of overlap in each of the Venn diagrams shows that the vast majority of SNPs were shared by all three plants within a given group (97.9% in YF, 98.9% in YC, and 99.6% in SC), indicating that the genetic diversity between individuals of a given group is very low. Within each group, Shandong plants had the fewest alleles associated with unshared SNPs (66 to 158) while field-grown Yukon plants had the most (328 to 678). We found no evidence that a single plant, whether in the field or the cabinet sample groups, had a disproportionate contribution of distinct SNP-associated alleles that were not shared by other plants in their respective group.

The genome-wide collection of polymorphisms that differentiate between the Yukon and Shandong *Eutrema* accessions given in Additional file 3 can be used to link allele frequency to traits associated with specific environmental conditions. Specifically, different accessions of *Eutrema* have been studied with the aim of identifying physiological and biochemical features associated with their adaptations to extreme environmental conditions [6,8,9,39]. With respect to the two accessions used in this study, the climate of Shandong Province is described as temperate and subject to monsoonal rains and that of the Yukon as semi-arid and subarctic. Thus the two accessions thrive on saline soils under otherwise contrasting environmental conditions, raising the prospect that the plants have common as well as distinct coping strategies to locally adapt to their respective habitats. As has been the case in studies of natural *Arabidopsis* accessions, the availability of SNPs that differentiate *Eutrema* accessions will facilitate the application of genetic approaches such as the analysis of recombinant inbred lines (RILs) or genome-wide association mapping of traits of interest [20].

### Patterns of global gene expression in *Eutrema* leaves

**Figure 2:**
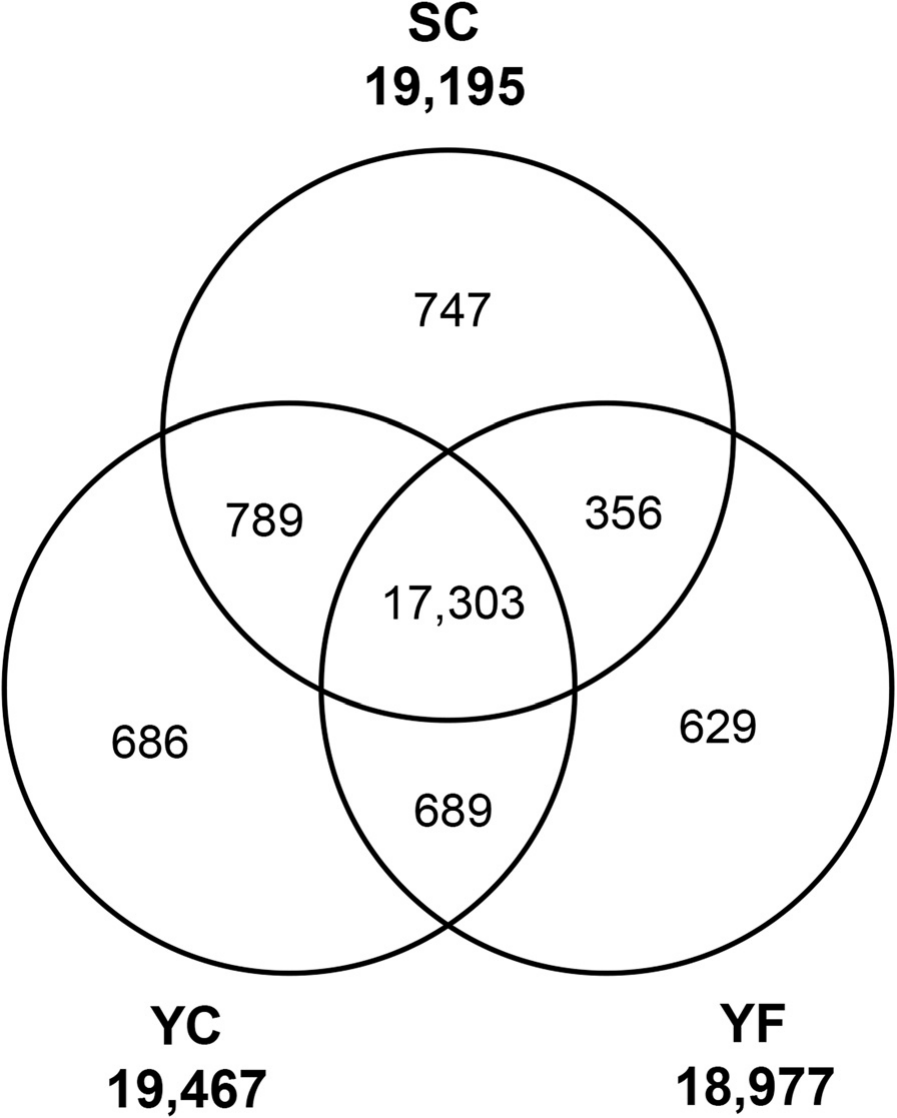
**Number of genes expressed in *Eutrema salsugineum *leaves.** Overlap in genes expressed between SC, YC, and YF plants is presented in Venn diagram form. The total number of genes expressed within each of the groups is indicated in bold text.

The number of expressed genes detected in each group was close, ranging from 18,977, in the YF group to 19,467 in the YC group (Figure 2). The majority of expressed genes (17,303 or 64%) were expressed in all groups while the proportion of genes that were only detected in a single group was low (from 2.3% in YF to 2.8% in SC samples). The expression levels of all *Eutrema* genes in this study can be visualized using a “*Eutrema* eFP browser” at the Bio - Analytic  Resource  [40]  (http://bar.utoronto.ca/efp_Eutrema/cgi-bin/efpWeb.cgi). The transcript abundance associated with a given gene can be queried using either a *Eutrema* locus identifier (e.g. Thhalv10012839m.g) or an *Arabidopsis* locus identifier (e.g. At5g49740).

**Figure 3:**
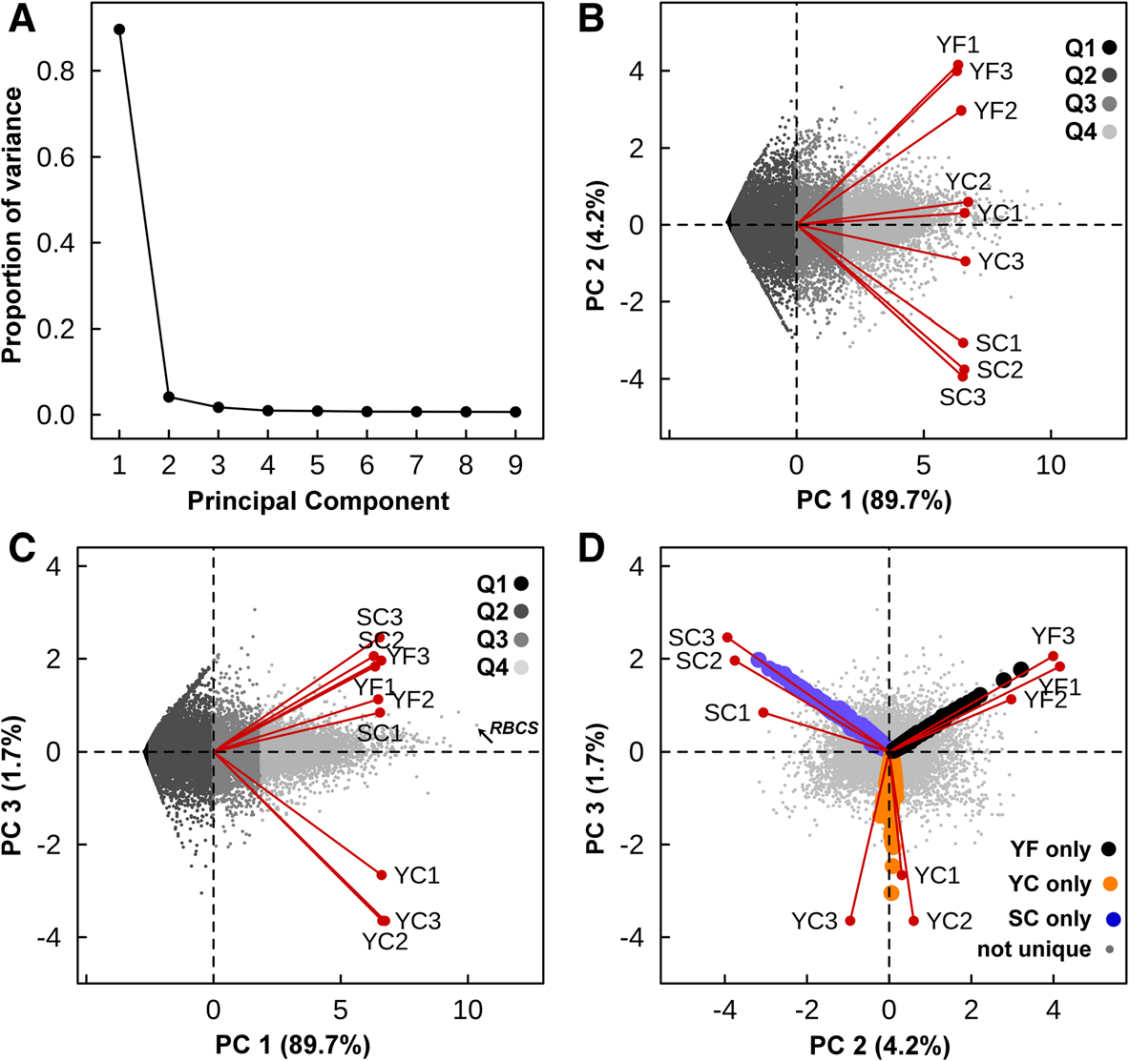
**Principal component analysis of global gene expression in nine *Eutrema *plants.** Expression estimates for 27,016 *Eutrema* genes in nine plants were log-transformed (see Methods) and subjected to PCA using a covariance matrix. **(A)** Scree plot illustrating the proportion of variance accounted for by each principal component. Scores and loading biplots of genes and cDNA libraries (red vectors) along principal components are shown in panels **B**, **C**, and **D**. **(B)** PC1 vs. PC2: Genes were divided into four equal bins (Q1 to Q4) according to their median expression level across all libraries and shaded to visualize relative expression level. Least abundantly expressed genes are black and most abundantly expressed genes are shaded light grey. **(C)** PC1 vs. PC3: Genes were divided into bins and indexed for expression level as described for **B**. **(D)** PC2 vs. PC3: ● gene expression detected only in YF plants; “orange circle symbol” gene expression detected only in YC plants; “blue circle symbol” gene expression detected only in SC plants. Genes represented by grey points were expressed in multiple plant groups.

With the majority of genes expressed by all of the plants (Figure 2), we expected that the expression patterns between the transcriptomes from the nine *Eutrema* plants would show considerable overlap. However, we also hypothesized that plants found at the field site would exhibit greater variability in their gene expression patterns relative to plants grown in a controlled environment chamber. To explore sources of variance within the *Eutrema* transcriptomes we used principal component analysis (PCA), in which observations were the nine plants and variables were the normalized gene expression estimates for each of 27,016 *Eutrema* genes. Briefly, in PCA axes called principal components (PCs) are identified. The first PC corresponds to the axis of greatest variation and all PCs, including PC1, describe sources of variation that are uncorrelated to each other. These uncorrelated axes are linear combinations of the original variables and can be used to reveal sources of variance in the data set. The scree plot in Figure 3A shows that the first three PCs explain 95.6% of the total variance in the dataset with PC1 contributing 89.7%, PC2 4.2%, and PC3 1.7%.

To interpret the biological significance of the PCs we examined the scores (red points), which are the values of each of the nine plant cDNA libraries (observations) (Figure 3B,C). These biplots contain both the scores for the observations and loadings for the variables (Figure 3B,C). This approach allows us to identify genes having expression levels (greyscale points) that are highly positively or negatively correlated with a given PC. The extent of correlation between the expression level of a gene and a PC is indicated by the magnitude of their loadings as shown by the values on the PC axes. PC1 explains the majority of variance in the dataset (Figure 3A). However, the scores for the nine plants for PC1 are similar. Expressed genes with high loadings for PC1 include Thhalv10019202m.g, the *Eutrema* orthologue of At5g38430, the locus encoding the ribulose bisphosphate carboxylase small subunit. Not surprisingly, *RBCS* was the most abundantly expressed gene in every library at an average expression level of 12,425 reads per kilobase per million mapped reads (RPKM). In contrast, genes with low loadings for PC1 are those showing no or low expression in all plants. Taken together, these loading and score patterns suggest that PC1 describes transcript abundance irrespective of the plant source. To explore this interpretation, the median expression level across all transcriptome libraries was determined for each of the 27,016 genes (see Additional file 6) and then the expression levels were divided into four equal bins based upon their magnitude relative to the median. Genes located in each bin were assigned a different gray-scale shade, producing a discrete gray-scale distribution pattern along the PC1 axis. Genes whose expression was not detected in this study are variables with the lowest loadings for PC1 while genes showing increasingly higher expression levels have progressively higher loadings for PC1 (Figure 3B,C).

The score and loadings biplot of PC2 vs PC3 is shown in Figure 3D. The scores for the plants within each group cluster together but each group has distinct associations with PC2 and PC3. Scores for YF plants are positive for both PC2 and PC3. SC plants score negatively on PC2 and positively on PC3. YC plants cluster around zero on PC2 and score negatively on PC3. Thus together, PC2 and PC3 may explain a relatively small fraction of the total variance in the genome-wide expression data (Figure 3A) but they capture the majority of variance that distinguishes the plant groups from each other. Moreover, the clustering of the three individual plants within each group relative to the PC2 and PC3 axes (Figure 3D) is significant in that it highlights the similarity in overall gene expression shown by individual plants comprising each group. While we expected that intentionally inbred, cabinet-grown plants would show highly correlated patterns of gene expression, we did not anticipate that plants randomly chosen from a field population in the Yukon (YF1, YF2, YF3) would be so similar with respect to this property (Figure 3D). The leaf transcriptomes obtained from individual plants in a wild population of *Eutrema* were as comparable to each other as those obtained from plants grown under protocols designed to control genetic and environmental variability and optimize reproducibility.

In contrast to PC1, the biological interpretation of the PC2 and PC3 axes is more complex given the large number of genes expressed, the low factor loadings associated with the majority of these genes and the fact that relatively few genes are strongly associated with PC2 and/or PC3. To address this challenge we used a variety of approaches to infer the biological meaning of PC2 and PC3. Thus we identified differentially expressed genes (DEGs) and used this information to group SC, YC, and YF plants on the basis of similar and contrasting patterns of gene expression. We also plotted the PC scores as norms of reaction plots and the DEGs as heat maps to facilitate comparisons between the transcriptomes and help identify and interpret patterns associated with the PCs and expressed genes. Finally, we categorized DEGs using GO slim terms in order to associate the gene products with putative biological functions.

### Accession- and location-associated patterns of gene expression in *Eutrema*

Our biological interpretation of the significance of PC2 and PC3 comes, in part, from examining the class of genes that were only found to be expressed in a single group of plants (Figure 3D). Loadings for these genes are highly correlated with the scores for each of the YF, YC, and SC plant groups. With transcriptomes obtained from individual *Eutrema* plants within each group being similar, we expected that meaningful patterns could be discriminated among the DEGs. We used DESeq [41] to identify DEGs in pairwise comparisons between the three groups of plants with the three transcriptomes of plants within each group treated as biological replicates. Gene expression estimates for all DEGs in each plant as well as their patterns of differential regulation are presented in the three tabs of Additional file 7.

**Figure 4:**
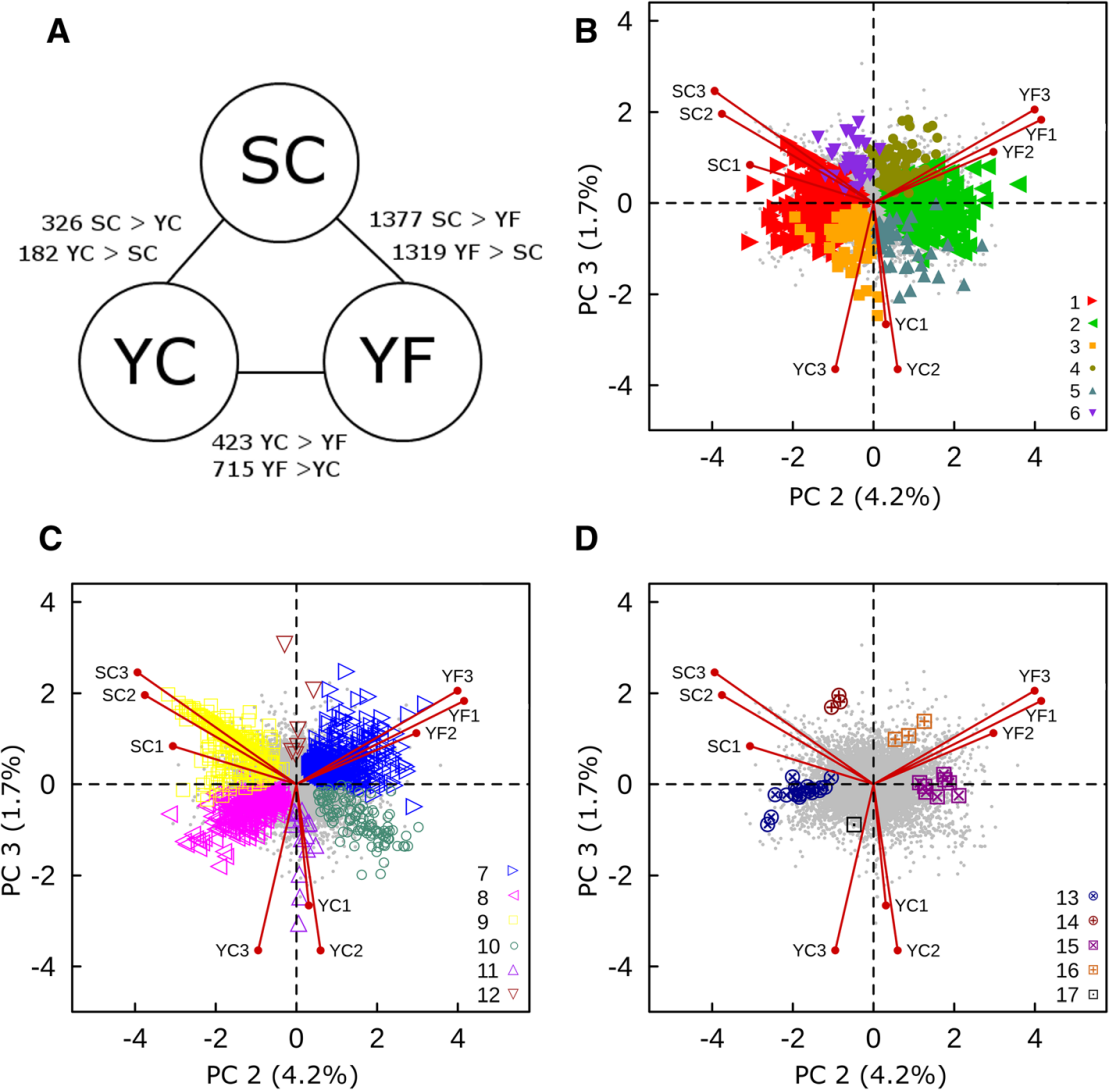
**Influence of genotype and location on *Eutrema *gene expression. (A)** Number of genes up- or down-regulated in pair-wise comparisons between Shandong cabinet (SC), Yukon cabinet (YC), and Yukon field (YF) plant groups. 2,989 DEGs are indicated on PCA biplot (PC2 vs. PC3) using the 17 categories described in Table 5. Genes not differentially expressed are plotted as grey points and DEGs are shown in color and grouped as follows: **(B)** Genes in Categories 1 to 6, which are differentially regulated between a single, pair-wise comparison of the three plant groups, **(C)** Genes in Categories 7 to 12, which are differentially regulated in two pair-wise comparisons and **(D)** Genes in Categories 13 to 17 representing genes differentially regulated in all three possible pair-wise comparisons between plant groups.

A total of 2,989 genes, or only 11.1% of the annotated *Eutrema* genome, were identified as differentially expressed (Table 5). The largest number of DEGs observed was between SC and YF transcriptomes (at 2,696 genes or 90% of all DEGs), a comparison involving genotype and location (Figure 4A). The number of genes differentially expressed between plant groups varying by genotype only was less than half (SC vs. YC = 508 genes or 17% of all DEGs) of the number distinguishing the plant groups that varied with respect to location but not genotype (YC vs. YF = 1,138 genes or 38% of DEGs).

**Table 5:** DEGs are categorized into discrete patterns of gene expression

**Category**	**Significant comparisons**	**Determinants for significance**	**Genes involved*(n)***	**Symbols for Figure**4**(B, C, D)**	
1	1	SC > YF	793	**▸**	Red
2	1	YF > SC	604	**◂**	Green
3	1	YC > YF	92	**■**	Orange
4	1	YF > YC	109	**●**	Green
5	1	YC > SC	40	▲	Blue
6	1	SC > YC	31	▼	Purple
7	2	YF > SC, YF > YC	586	**▹**	Blue
8	2	SC > YF, YC > YF	297	**◃**	Magenta
9	2	SC > YC, SC > YF	265	■	Yellow
10	2	YC > SC, YF > SC	118	**◯**	Blue
11	2	YC > SC, YC > YF	15	△	Purple
12	2	SC > YC, YF > YC	6	**▽**	Brown
13	3	SC > YC > YF	18	**⊗**	Blue
14	3	SC > YF > YC	3	**⊕**	Red
15	3	YF > YC > SC	8	**⊠**	Magenta
16	3	YF > SC > YC	3	**⊞**	Brown
17	3	YC > SC > YF	1	**⊡**	Black

The DEGs presented in Figure 4 are sorted into 17 distinct categories according to the significance of differential gene expression between the three groups of plants. For instance in Category 1, expression of 793 genes was significantly higher in SC plants than in YF plants but expression of these genes was not significantly different between SC and YC or between YC and YF.

The 2,989 DEGs were assigned to one of 17 non-overlapping categories following pair-wise comparisons between the plant groups (Table 5). Most of the genes showed a statistically significant expression difference from a single, pair-wise comparison between two of three plant groups (1,669 genes; Categories 1 to 6 on Table 5). The second most frequent scenario was genes whose differential regulation proved significant in two pair-wise comparisons (1,287 genes; Categories 7 to 12 on Table 5). A third, smaller set of genes (33), had transcript expression levels for each of the plant groups that were significantly different from each other (Categories 13 to 17 on Table 5).

Our PCA analysis shown in Figure 3D indicated that PC2 and PC3 best described the sources of variance between the three plant groups. In Figure 4B-D the PC2 versus PC3 biplots are redrawn with genes highlighted using colored symbols representing their DEG categories (Table 5). The DEGs shown on the biplots are not randomly distributed in that symbols relating to the different categories are grouped in discrete zones around the origin. This organization is best exemplified by the zonation seen in Figure 4C, showing the DEGs of Categories 7 through 12. The up-regulated genes of YF relative to both SC and YC (Category 7 ) have positive loadings on both PC2 and PC3 axes. In contrast, the down-regulated genes of YF relative to the other samples (Category 8 ) have negative loadings for both PC2 and PC3. This arrangement of DEGs around the origin is recapitulated for the remaining Categories (9  and 10 ◯, 11 and 12). Categories 11 and 12 describe genes that are differentially regulated in YC plants relative to both SC and YF plants and their loadings are associated primarily with PC3 and negligibly with PC2. Figure 3D reiterates the association between YC and PC3 in that genes expressed only in YC plants and genes more highly expressed in YC1, YC2, and YC3 plants are all strongly associated with PC3.

Analogous observations provide insight into the variance associated with PC2. In Categories 1  and 2 (Figure 4B), DEGs are defined by significant expression differences between YF and SC plants. In Figure 4D (Categories 13  and 15 ), YF and SC transcript expression is significantly different whereas the expression level for genes in YC plants is intermediate between YF and SC plants. In both of the above instances PC2 explains a source of variance in gene expression between the SC (more negative PC2) and YF (more positive PC2) transcriptomes that is not influenced by YC transcriptomes. Conversely, PC3 explains a source of variation uncorrelated to PC2 that describes differences in gene expression in YC plants. Both the zonation of DEGs around the origin of PC2 and PC3 (Figure 4B-D) and the distribution of the scores, including their correlation within plant groups, emphasizes that PC2 and PC3 capture the main sources of variance between the plant groups despite their minor contribution (5.9%) to the total amount of variance in the dataset.

**Figure 5:**
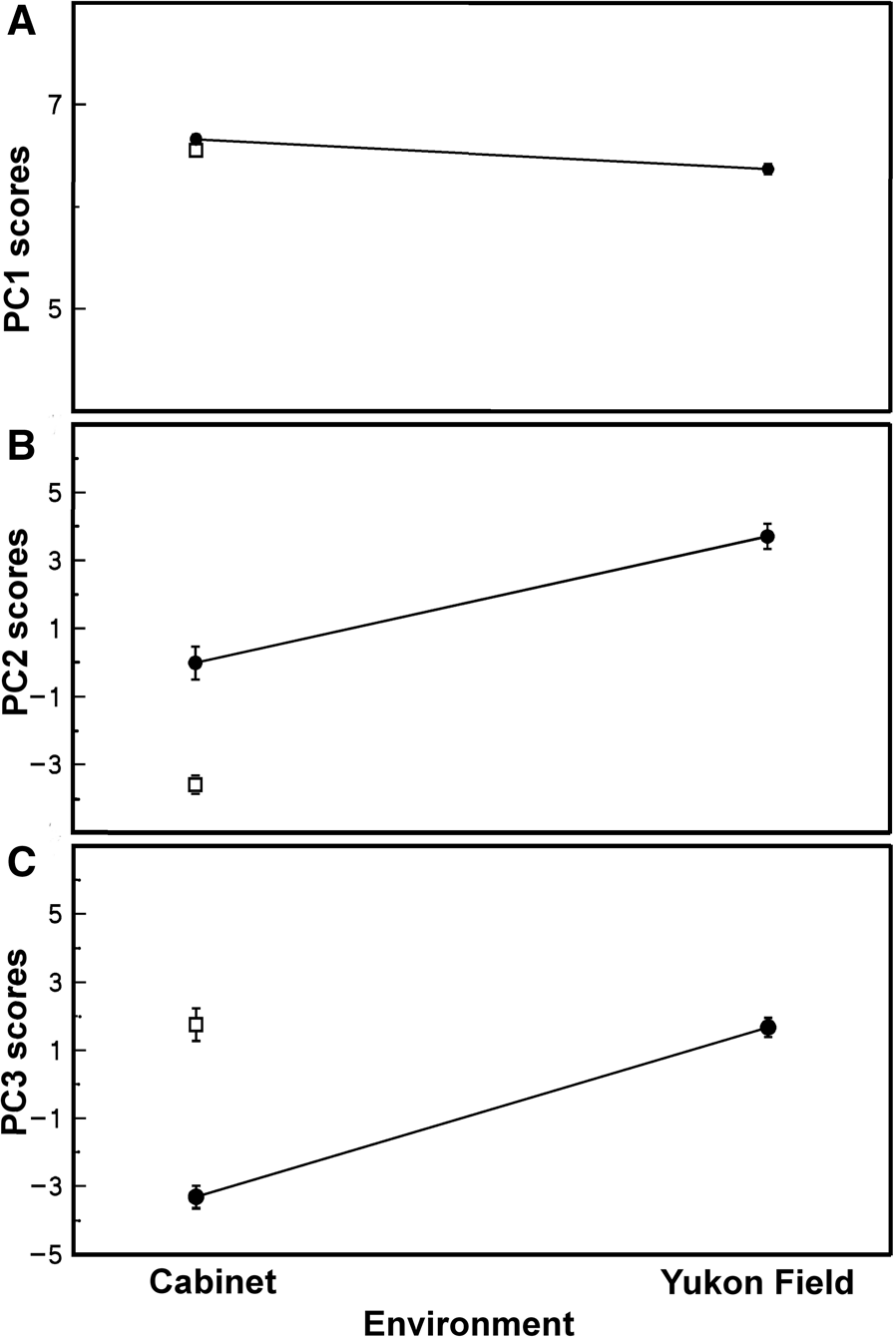
**Norm of reaction plots of PC scores. (A)** PC1 scores, **(B)** PC2 scores and **(C)** PC3 scores are shown as a function of environment. The means ± SE of the PC scores for each of the 9 libraries are plotted as a function of growing environment, cabinet and Yukon field. Shandong (⊡), Yukon (●).

The associations between PC scores and transcriptome source can also be displayed as norm of reaction plots (Figure 5), with measures for the Yukon plants in growth cabinet and Yukon field, and measures for Shandong plants in growth cabinet. The plot of PC1 scores (Figure 5A) shows no variability among the nine genome-wide transcription profiles tested. This demonstrates the lack of genetic variability between accessions or in response to the environment between transcriptomes with respect to PC1. In contrast, plots of PC2 and PC3 scores show differences in transcriptome patterns between the plants within the cabinet indicating genetic differentiation, between accessions and between the Yukon plants grown in the field and Yukon plants grown in the cabinet, indicating phenotypic plasticity. For PC2, SC and YF scores were the most different with YC scores intermediate of SC and YF. PC2 scores from transcriptome profiles of SC and YC plants grown under common cabinet conditions are significantly different, indicating the two accessions differ genetically in the gene expression patterns contributing to PC2. However, PC2 scores also differ in global transcription patterns between YC and YF samples indicating that Yukon plants show plasticity in gene expression associated with PC2 in response to the environment. Interestingly, for PC3 the SC and YF scores are the same but YC and YF scores are significantly different, as are those for SC and YC. Thus PC3, like PC2, shows genetics as a source of variation between SC and YC transcriptomes, but many expressed genes with high loadings on PC3 do not differ between SC and YF samples. Additional file 8 is a list of the 50 expressed genes with the greatest loadings contributing to the negative position of YC scores relative to YF and SC scores for PC3. A common attribute of genes on this list is that they show comparatively higher average transcript levels for YC samples relative to the low (or no) detected expression for the same genes in YF and SC samples.

Phenotypic plasticity has been reported for Yukon field and cabinet plants with field plants largely void of rosette leaves and cabinet plants rarely having cauline leaves [7]. This morphological difference between field and cabinet phenotypes precludes selective sampling of either cauline or rosette leaves from both sources. Thus the differences seen between YC and YF gene expression shown by PC3 could arise from developmental differences between cauline leaves of field plants and rosette leaves of cabinet-grown plants. To address this possibility, we prepared a transcriptome from cauline leaves of flowering bolts removed from cabinet grown Yukon plants. This sample was equivalent to the cabinet cauline leaf cDNA used as a control in comparisons with field samples for the microarray study reported by Guevara et al. [7]. Additional file 8 includes a column with transcript levels found for YC cauline leaves for comparison to the YC and YF expression values. For 38 of the 50 genes on this list, the transcript data shows that YC rosette and cauline leaves from cabinets share the same pattern of higher gene expression relative to YF and SC samples. This observation supports the proposal that PC3 describes variation in gene expression of Yukon leaves of cabinet-grown plants (cauline or rosette) and that these genes are not co-expressed to nearly the same extent in leaves of SC plants in the same cabinet or Yukon plants in the field. The biological significance of the genes comprising this list is difficult to determine as many have either a poorly defined role in plants or no known biological function.

PCA of the entire expression dataset, comprised of 27,016 genes in all nine samples, demonstrated that transcriptome profiles of the naturally occurring Yukon plants were as similar to each other and as reproducible as the profiles of the chamber-grown plants (Figure 4B-D). Using the smaller subset of transcriptionally variable genes we assessed the extent of variability in DEG expression among plants within and between sample groups. We anticipated that more irregularities in the expression of DEGs might be observed in the field-grown plants compared to the plants grown in controlled conditions. Expression estimates for each of the 2,989 DEGs within the nine libraries were subjected to hierarchical cluster analysis (HCA) to visualize their expression patterns within and between groups.

**Figure 6:**
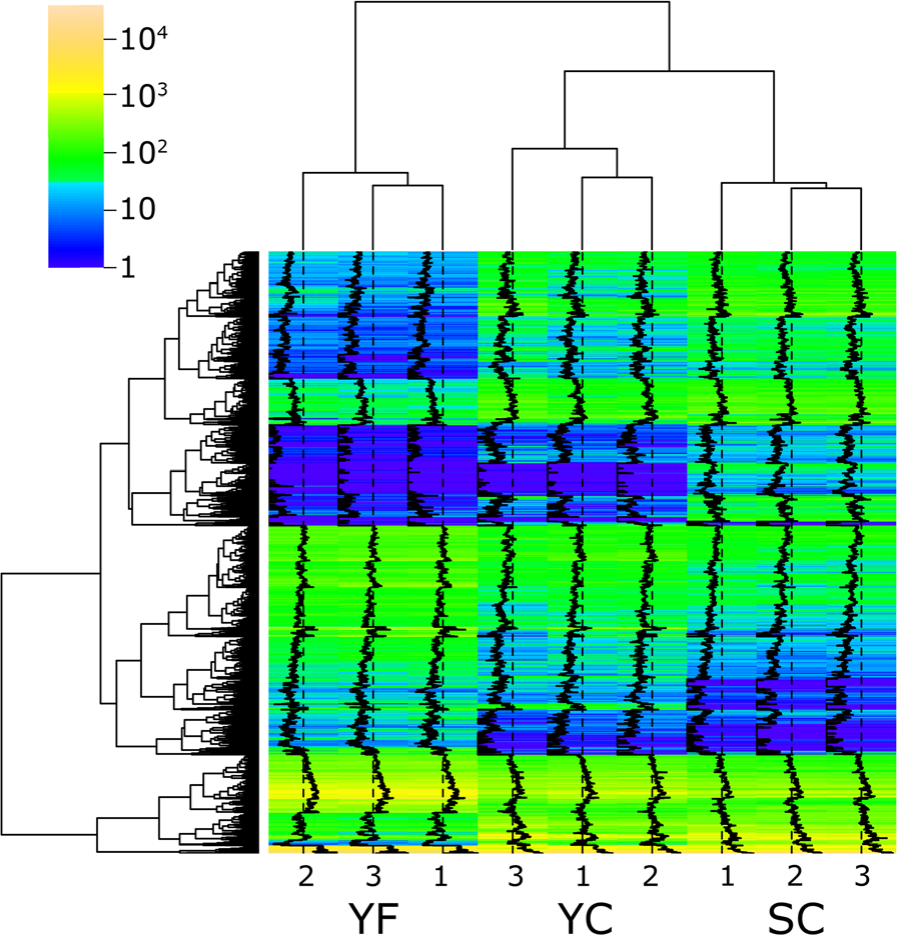
**Hierarchical clustering of genes differentially expressed in *Eutrema*.** Heat map of 2,989 differentially expressed genes generated using complete-linkage clustering of Euclidean distances between log-normalized expression values. Expression levels for genes in each cDNA library are represented as colors ranging from purple (little or no expression) to yellow (abundant expression). On the heat map, expression levels of DEGs in each library are also illustrated by black traces. Clustering at the level of individual libraries is represented by the dendrogram at the top of the heat map and clustering at the level of individual genes is presented at the left of the heat map. All nodes in the dendrogram had bootstrap probability and approximately unbiased *p-*values greater than 0.99.

The dendrogram at the top of the heat map shown in Figure 6 groups together the datasets obtained from replicate plants with the expression profiles from cabinet-grown plants (YC and SC) sharing the same node. The HCA shows that the sample group clustering separately from the SC and YC plants is the YF group, indicating that there are fewer correlations between the transcript abundance of DEGs in cabinet and field-grown plants than between the cabinet-grown plants of the two accessions (Figure 6). This interpretation is also supported by our observation that a greater number of genes were identified as differentially expressed when YC and YF transcriptomes were compared (1,138) versus the SC and YC transcriptome comparison (508) (Figure 4A).

The heat map of Figure 6 also includes “traces” indicating the normalized, log transformed expression level associated with each DEG. The traces show discernible differences in gene expression between the three groups of plants but they produce a nearly identical pattern within each of the groups. The heat map provides a visual means to demonstrate that expression of DEGs was very consistent within each group of three plants. Thus while the plants at the field site may show a pattern of gene expression that distinguishes them in a cluster apart from the cabinet-grown plants, the traces associated with DEGs from the YF sample group show an internally consistent pattern of expression despite having been selected randomly from a single natural site in their native Yukon habitat. As discussed earlier, the Yukon field and cabinet plants show plasticity with respect to predominance of cauline and rosette leaves, respectively, so the array of differentially expressed genes between field and cabinet Yukon plants could represent differences between leaf type. Additional file 1: Figure S2 is a version of the HCA shown in Figure 6 that has been modified to include the cabinet-derived Yukon cauline leaf expression data corresponding to the 2,989 DEGs distinguishing the SC, YC, and YF transcriptomes from each other. The gene expression data associated with the cauline leaves from Yukon cabinet-grown plants are grouped in the same clade with the YC rosette leaves and apart from the cauline leaves of YF plants or rosette leaves of SC samples. This outcome is consistent with the results of Ma et al. [41] who used microarrays to compare expressed genes representing various organs of *Arabidopsis*. They show that cauline and rosette leaves collected from plants of varying age but grown under identical conditions of temperature, light intensity and duration have similar expression profiles with their corresponding datasets sharing the same clade as determined by average-linkage clustering with correlation distance analysis of relatedness. Thus we conclude that the pattern of DEGs given in Figure 6 represent differences attributable to variable genotype and/or environment and not different leaf types from plants of the same accession grown under comparable environmental conditions.

### Biological significance of differentially expressed genes in *Eutrema*

**Figure 7:**
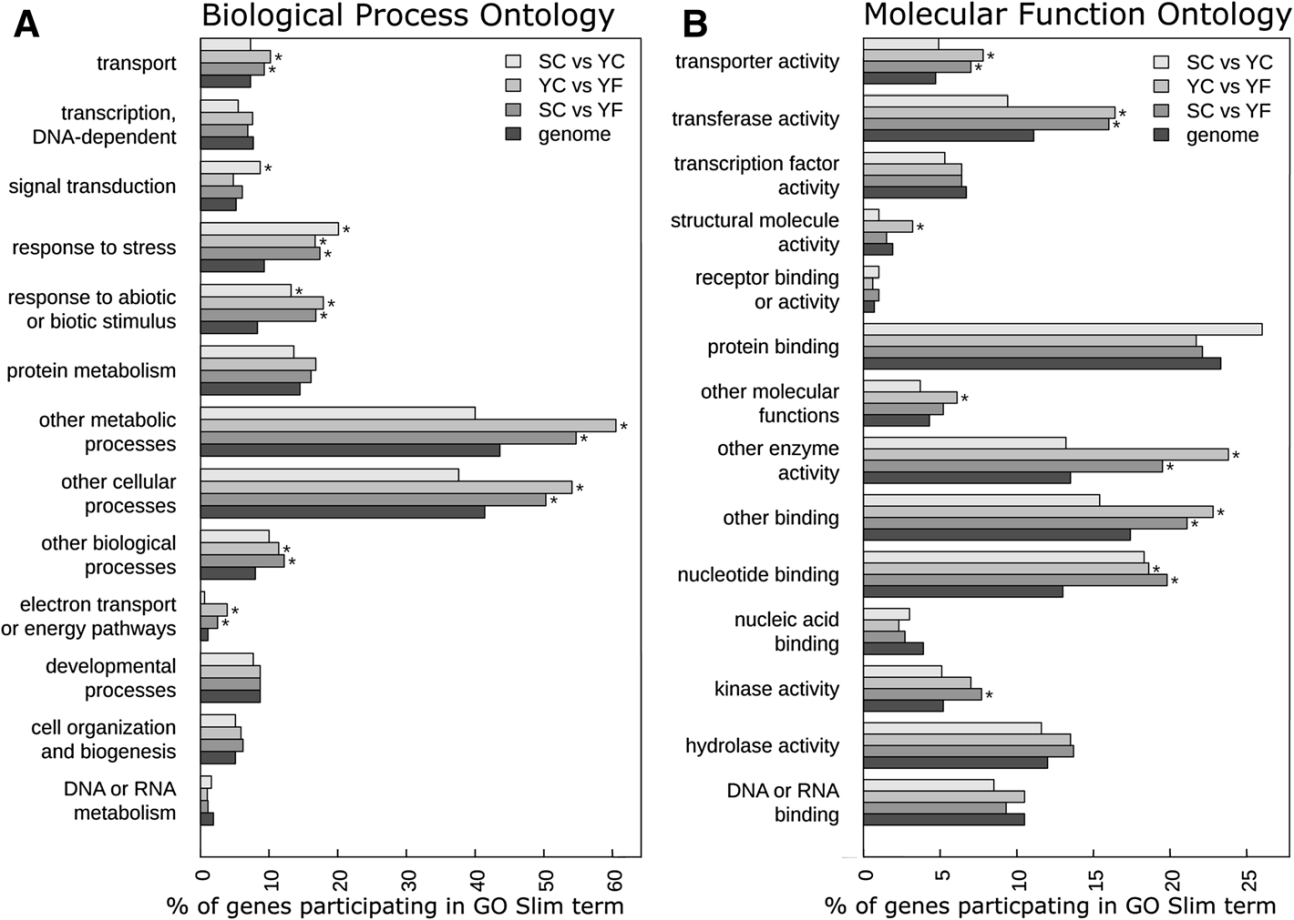
**Gene Ontology enrichment analysis of differentially expressed genes in Yukon and Shandong *Eutrema.*** GO enrichment was performed on the set of differentially expressed genes identified in each of the three pair-wise comparisons between SC, YC, and YF plant groups for **(A)** “Biological Process” and **(B)** “Molecular Function”: ontologies. GO categories in which the proportion of participating differentially expressed genes is significantly different (*p* < 0.05) from the proportion expected of all 27,016 annotated genes in the genome is indicated by an asterisk (*).

Using the extensive similarity between *Arabidopsis* and *Eutrema* genes we examined the predicted biological functions represented by the 2,989 genes identified as differentially expressed in comparisons between SC, YC, and YF sample groups using a GO analysis. In the first approach we identified a number of GO slim categories that were significantly over- or under- represented in comparisons made between the sample groups (Figure 7). For reference we also determined the predicted frequency of gene products in a given GO category using the entire *Eutrema* genome (27,016 genes). The result of this GO analysis shows that a broad array of biological process (Figure 7A) and molecular function (Figure 7B) associated products are represented by the DEGs identified through our pair-wise comparisons. Only two GO slim terms in the ontology domain relating to biological process showed a significant over-representation of genes for all three pair-wise comparisons (SC-YC, SC-YF, YC-YF) relative to the whole genome and these were “Response to stress” and “Response to abiotic or biotic stimulus” (Figure 7A).

We next identified GO terms that were over-represented by the DEGs. Using a cutoff of Q ≤ 0.05, there were 7 and 135 GO terms describing DEGs between SC versus YC and YC versus YF transcript comparisons, respectively. The GO terms showing a high degree (Q ≤ 10^-10^) of over-representation are shown in Table 6 and again the term “Response to stress” is common to both comparisons. For plants representing the two accessions growing under identical conditions, namely SC and YC plants, DEGs associated with the GO term “Defense response” were the most highly over-represented group. In contrast, for the comparison involving the same accession in different environments (YC and YF) the GO term that was most significantly enriched by DEGs was “Photosynthesis”.

**Table 6:** **Functions associated with *Eutrema *genes differentially expressed according to accession or location**

**Shandong cabinet vs. Yukon cabinet**			**Yukon field vs. Yukon cabinet**		
**GO term**	**GO ID**	**Q value**	**GO term**	**GO ID**	**Q value**
Defense response	0006952	1.67 × 10^-13^	Photosynthesis	0015979	8.58 × 10^-23^
Response to stress	0006950	6.33 × 10^-10^	Metabolic process	0008152	1.33 × 10^-15^
			Oxidation-reduction process	0055114	1.37 × 10^-13^
			Response to stimulus	0050986	1.37 × 10^-13^
			Photosynthesis-light reactions	0019684	3.73 × 10^-12^
			Response to abiotic stimulus	0009628	1.33 × 10^-11^
			Response to biotic stimulus	0009607	2.73 × 10^-11^
			Response to other organism	0051707	6.17 × 10^-11^
			Response to stress	0006950	6.95 × 10^-11^
			Response to bacterium	0009617	2.34 × 10^-10^

GO terms that are significantly enriched are presented. Q values show the level of significance for each comparison within the indicated plant groups.

GO classifications offer a means to explore transcriptome data and it can provide insight into the functional significance of a gene or group of genes. We have no evidence that the cabinet-grown plants (SC and YC) were exposed to pathogens so the biological significance of the gene products associated with the shared GO term “Defense response” is difficult to evaluate. On the other hand, the GO term “Photosynthesis” is not an unexpected category to find differentially expressed genes given the dissimilar light conditions between the Yukon field site and the cabinet. Indeed, a key difference between the environment experienced by the YF and YC or SC plants was light intensity, measured as 1400 μmol m^–2^ s^–1^ at mid-day at the field site when plants were collected [7] and a constant 250 μmol m^-2^ s^-1^ for a 21 h day in the growth chamber. Given this almost six-fold difference in irradiance, it is not surprising that “Photosynthesis” was the most over-represented GO term in the comparison between field-grown and cabinet-grown Yukon plants, and the most statistically significant category in the entire GO dataset.

Of 200 *Eutrema* genes that were found to be associated with the GO term “Photosynthesis” (GO:0015979), 71 show significant differences following pair-wise comparisons between SC, YC, and YF plants (Additional file 9). If we confine our discussion to differences involving the YC and YF plants, there are only 52 genes relevant to this comparison. Interestingly, in every case the transcript abundance associated with YF plants exceeds that of YC plants (YF > YC) with the greatest fold change (ca 13-fold) found for a gene identified as ferric reduction oxidase 7 (orthologous to At5g49740); a representative from the small number of DEGs comprising the YF > SC > YC Category of Table 5. Predictably, genes whose products have a longstanding association to photosynthesis are on this supplementary list (Additional file 9) including the small subunit of ribulose bisphosphate carboxylase (At5g38410), the delta subunit (At4g09650) and gamma subunit (At4g04640) of the chloroplast ATP synthase and various light harvesting pigment proteins (Psb29, LIL3.2, PsbP to name a few). Interestingly, the genes whose transcripts consistently show an approximately three-fold or higher level of expression in the field relative to the cabinet encode many products associated with the chloroplast NAD(P)H complex (NDH), involved in photosystem 1 (PSI) photosynthetic cyclic electron transport and chlororespiration (reviewed by [42]). These products include NDH subunits NDF2/NDH45 (At1g64770), NDF4 (At3g16250), NDF6 (At1g18730), PPL2 (At2g39470; PsbP-like2, implicated in the repair of the NDH complex), chlororespiratory reduction 1 (CCR1; At5g52100), CRR3 (At2g01590) and CRR23 (At1g70760). The NDH associates with PSI in an interaction that is believed to involve LHCl (light harvesting chlorophyll) subunits Lhca5 and Lhca6, encoded by orthologues of At1g45474 and At1g19150, respectively [42]. The genes associated with these minor components of PSI both show statistically significantly higher transcript levels in field plants relative to chamber grown plants (YC or SC). PsbQ and PsbP proteins are also regarded as integral to NDH complex accumulation and transcripts associated with these products are also up-regulated in the field. A hypothesis consistent with these observed differences in transcript abundance is that the field plants have a heightened capacity for PSI cyclic electron transport that uses a chloroplast NDH-dependent route. The NDH complex represents one route for cyclic electron transfer around PSI in *Arabidopsis*. There is evidence that the NDH complex exerts its main effects under stress where it is believed to prevent the over-reduction of PSI electron acceptors, reduce the generation of reactive oxygen species and, in general, provide protection from photoinhibition (reviewed in [43-46]). Statistically significant associations between stress and DEGs were reiterated in comparisons between YF and YC transcriptomes in the “Response to stress” and “Response to abiotic stimulus” categories (Table 6). The stress-responsive differences supported through GO analyses are all the more convincing given the changeable and stressful conditions at the field site compared to the growth chambers [7].

Light intensity at the field site can be variable but a far more consistent natural feature of the Yukon habitat where *Eutrema* grows is the presence of a highly saline soil. We previously reported that both the soil and *Eutrema* leaf tissue obtained from the field site contain high levels of Na^+^[7] and the turgid leaves exhibited low solute potentials consistent with plants having undergone osmotic adjustment [7]. We therefore expected that GO terms concerned with responses to salt exposure would also emerge as statistically significant when characterizing the transcriptional difference between the field-grown plants and unsalinized cabinet-grown plants. In support of this expectation, we identified 490 *Eutrema* gene orthologues participating in the “Response to salt stress” (GO:009651) category (see Additional file 10). However, this category was not identified as a statistically significant difference in any of the comparisons between plant groups. Of the 490 genes with some salt association, only 35 (7%) were differentially regulated in the comparison between YF and YC transcriptomes (19 genes up-regulated and 16 genes down-regulated in YF relative to YC). Thus only a small number of genes classically categorized as salt responsive were differentially regulated in Yukon plants exposed to a saline environment in the field. While this outcome may be surprising, it is entirely consistent with microarray experiments showing a smaller transcriptional change in *Eutrema* plants exposed to salt compared to other stresses [7,47,48]. It is possible that the actions of a few genes may orchestrate a large effect but, equally, it has been proposed that *Eutrema* genes required for salinity tolerance are constitutively expressed in this plant which is adapted to saline soil conditions [48,49] and therefore salt-responsive genes are not detected as DEGs. Regardless of the explanation, in contrast to the salt-inducible nature of many genes in *Arabidopsis*, the response of *Eutrema* to salt is associated with the altered expression of relatively few genes as determined by both microarray and RNA-Seq approaches.

## Conclusions

Due to its natural adaptations to harsh environments, *Eutrema salsugineum* has emerged as an important model for deciphering mechanisms of abiotic stress tolerance in plants [8]. One methodology employed by our group as well as others has been phenotypic, physiological and gene expression profiling of *Eutrema* in response to simulated stress treatments in growth cabinets [47,48,50,51]. Experiments of this type offer certain advantages, including the ability to select or manipulate the genotype of the plants studied, apply stress treatments in a prescribed manner and replicate experiments under tightly controlled environment conditions. However, recent reviews have highlighted weaknesses in this approach, namely that growth chambers do not reproduce the complexity and dynamic nature of a natural environment [52,53]. Nonetheless, natural environments are precisely the conditions in which traits conferring stress tolerance must manifest themselves. The unexpectedly low content of the osmoprotectant proline in leaves of field plants was discussed earlier as one such anomalous response between field plants on saline soil and cabinet plants irrigated with salt [7]. With these considerations in mind, perhaps it is not surprising that few genes identified as key candidates for abiotic stress improvements as a result of growth cabinet studies have been used successfully to improve stress tolerance under actual field conditions [52].

In this study we sought to determine whether the sensitivity and capacity for genome-wide resolution in gene expression offered by RNA-Seq approaches could be satisfactorily deployed in the investigation of a native plant exposed to stressful conditions in its natural environment. For this approach we had to consider the possible influence of natural genetic and environmental variation on gene expression that could interfere with, or even prevent, the detection of traits of interest. This concern has received little attention in the literature. Existing gene expression profiling of plants growing in field plots [54-56] or in a natural habitat [7] has reflected the pooled contribution of multiple individuals, thereby masking the extent of natural variation that is actually present. Our results show little evidence for genetic diversity among the natural plants sampled in the Yukon, thereby ruling out genetic variation as a major source of transcriptional variation in these plants. Furthermore, RNA-Seq transcriptomes obtained from three, individual plants randomly selected at the field site were as similar to each other as those obtained from intentionally inbred chamber-grown plants, demonstrating that natural environmental variation did not impede the identification of genes showing similar patterns of expression. This agreement is particularly striking given the extent of phenotypic plasticity displayed by this species but perhaps not surprising in light of recent evidence that *Eutrema* has a core set of platform independent traits in addition to those showing plasticity [7,51]. Moreover we show, using a Yukon population of *Eutrema*, that a physiologically relevant record of exposure to a natural habitat can be captured and retrieved from the transcriptomes of relatively few individual plants. This RNA-Seq data is publicly available and can be queried using the BAR eFP browser, which will facilitate the study of environment- or accession-associated differences in gene expression. Gene expression profiles from an extremophile plant in its natural habitat provide an invaluable platform from which sophisticated data mining followed by physiological and genetic approaches can be used to identify and test the adaptive significance of plant stress responses.

## Methods

### Plants and growth conditions

Cauline leaf tissue was harvested from mature, flowering Yukon *Eutrema* at a field site near Whitehorse, Yukon in 2005 as described in Guevara et al. [7]. Leaf tissue from individual plants was transferred to cryovials, flash frozen in liquid nitrogen, then transferred to a charged MVE XC20/3 V vapour shipper (Jencons Scientific, Bridgeville, PA) where samples were kept frozen at −150°C for transport to McMaster. Tissue was stored at −80°C pending analysis.

For growth cabinet studies, both Yukon (sourced from the field site in 2002) and Shandong (obtained from the Bressan lab, Purdue University) *Eutrema* accessions were subjected to single-seed descent (Yukon - 5 generations, Shandong - 4 generations) in an attempt to increase genetic homozygosity of the plants. Seeds were sterilized in 30% bleach, 50% ethanol and 0.1% Tween-20, rinsed in water and then mixed with 0.1% (w/v) Phytagel (Sigma) and pipetted onto a moistened soil mixture containing six parts Promix BX (Premier Horticulture, Rivière-du-Loup, PQ) and one part Turface (Profile Products LLC, Buffalo, NY) in individual 5 × 5 × 7 cm pots. Seeds in pots were stratified for either 2 days (Yukon) or 5 days (Shandong) at 4°C before transfer to a growth chamber (AC 60 Econair, Winnipeg, MB) set with a 21 h day and irradiance of 250 μmol m^-2^ s^-1^ and 22°C/10°C day/night temperature regime. Plants were watered daily as needed and fertilized one time per week with 1 g L^-1^ 20-20-20 (N-P-K) fertilizer. Of six cabinet-grown plants described in this work, all three Shandong plants and two Yukon plants were of the single seed descent lines described above and grown simultaneously in an AC 60 growth chamber in 2010 as described. The remaining Yukon plant was grown in the chamber in 2006 and was derived from a heterogeneous mixture of seeds maintained as a bulked pool from plants originally harvested at the Yukon field site in 2002. The last fully expanded pair of rosette leaves was harvested from 4 week-old cabinet-grown plants of both genotypes, flash frozen in liquid nitrogen and stored at −80°C until further analysis.

### Total RNA isolation and mRNA purification

Extractions of nucleic acids from *Eutrema* proved to be unsatisfactory using methods commonly employed for *Arabidopsis*. These issues were circumvented using a modification of the method described for cotton leaves in Wan and Wilkins [57]. Briefly, approximately 0.5 g of frozen leaf tissue in liquid nitrogen was ground to fine powder in a mortar and pestle and RNA was extracted according to the manufacturer’s recommendation with 8 mL Tri Reagent (Sigma). This first crude preparation of total RNA was resuspended in 1 mL nuclease-free water and purified of polyphenolics and carbohydrates using a “hot borate” step (200 mM sodium borate decahydrate, 30 mM Na-EGTA, 1% (w/v) SDS, 1% (w/v) sodium deoxycholate, 2% (w/v) polyvinylpyrrolidone (PVP 40000), 0.1% (w/v) DEPC, pH 9.0) followed by sequential RNA precipitations with 2 M lithium chloride then ethanol. From approximately 150 μg of this high quality total RNA, mRNAs were purified in 3–4 repeated rounds of oligo-dT selection using a Sigma mRNA miniprep kit (MRN-10). Abundance and quality of RNA following total RNA extraction was assessed using RNA 6000 Nano chips (Agilent) on a Bioanalyzer 2100 instrument and mRNA selections were continued until no rRNA peaks were visible on Bioanalyzer electropherograms.

### Sequencing library preparation

Procedures for RNA fragmentation, cDNA synthesis, sequencing adaptor ligation and size selection closely followed the Roche cDNA Rapid Library Preparation Manual, December 2010. Briefly, 200 ng of mRNA was chemically fragmented with ZnCl_2_ and used as a template to synthesize double-stranded cDNA using Roche Primer “random”. The product of each cDNA synthesis was end-repaired and indexed with the regular Rapid Library Adaptor with one exception: Libraries SC2 and SC3 were indexed by ligation of Roche MID Adaptors 4 and Adaptors 5, respectively. Each cDNA library was quantified by fluorometry on a Biotek Synergy 2 fluorometric plate reader. Size selection of each library in the 600–1200 bp range was verified by running a small aliquot on a High Sensitivity DNA chip (Agilent) on the Bioanalyzer 2100. All sequencing libraries were in the range of 10^8^ to 10^9^ molecules/mL and stored in this concentrated form in TE buffer at −80°C for several days to approximately 1 month until further analysis. Quantitative real-time RT-PCR was conducted on a Bio-Rad CFX96 instrument using primers specific for *Eutrema ACTIN1* (F - ACAGGGTGCTCTTCAGGAGCGAT; R - GCATGGTGTTGTGAGCAACTGGG), whose product spans an intron. Contaminating genomic DNA was not detected in any sequencing library.

### Emulsion PCR and titration of sequencing libraries

Small volume emulsion PCRs (SVemPCRs) were performed to determine the optimum library-to-bead ratio for clonal amplification of each sequencing library with methodology closely following the Roche emPCR Method Manual Lib-LSV, October 2009 (version 2). Each cDNA library was titrated using 2, 4 and 8 cDNA molecules per bead. A Qiagen Tissuelyser II was used to prepare emulsions and amplifications were performed on a Biorad iCycler instrument. Following SVemPCR, emulsions were broken and DNA-capture beads carrying single-stranded cDNA were enriched and counted using a Z1 Coulter Counter (Beckman Coulter) following Roche instructions. For each library, the amount of cDNA required to produce a 10% enrichment of sstcDNA containing beads was estimated and used as input for large volume emPCR. One to two large volume emPCR reactions for each library were performed according to the Roche emPCR Method Manual - Lib_L LV, October 2009. The vacuum-assisted technique for breaking emulsions and bead recovery was followed. For each library, large volume emPCR reactions resulting in 8-15% bead enrichment and a total number of sstcDNA containing beads exceeding 3.5 million were used in subsequent sequencing.

### 454 GS FLX titanium sequencing

Roche GS FLX Titanium sequencing kits were used for all libraries, which were sequenced at a depth of one complete PicoTiterPlate per library with one exception: Multiplexed libraries SC2 and SC3 were sequenced together on a single plate. A total of 3.5 to 4 million sstcDNA containing beads were loaded onto each picotitre plate. 454 pyrosequencing and subsequent image analysis followed default parameters suggested by Roche.

### Bioinformatics

#### Quality control - trimming

Removal of adaptor sequences and initial quality trimming was performed using GS *De Novo* Assembler v2.6 using default parameters. Sequences were trimmed further using SeqTrim v0.110 [58] with a minimum quality value of 20, a window size of 10 and no contamination removal step. Reads less than 80 bases in length were excluded from further analysis.

#### Read alignment and assembly

Trimmed reads were aligned to the *Eutrema* genome release (http://www.phytozome.net/thellungiella) [14] using the splice-aware aligner GMAP v2011-11-30 with default parameters [18]. Alignments were separated into uniquely mapped, non-uniquely mapped, and unmapped sets using a custom Perl script. The existing genome annotation was enriched by predicting genes using the Cufflinks package v1.3.0 [23,59]. Uniquely mapped reads from each replicate were assembled independently using the reference annotation based transcript (RABT) assembly method using Cufflinks. Cuffmerge was used with and without RABT assembly to generate two alternative merged annotation files. Predicted genes from each annotation file that do not overlap with annotated genes were identified using intersectBed from BEDTools v2.13.4, enforcing same-strandedness [60]. These two sets of non-overlapping genes were then compared with intersectBed to produce the final filtered, non-redundant list of Cufflinks predicted genes.

#### Enrichment of gene annotation

Protein blast databases were constructed for the TAIR version 10 *A. thaliana* annotation [61] as well as the Phytozome v7.0 *A. lyrata* annotation [62], *E. parvulum* v2 (http://www.thellungiella.org/data) [24], and RefSeq plants (http://ftp.ncbi.nlm.nih.gov/refseq/release/plant/ on 2012-03-06). The best blastx hits (E ≤ 1^-10^, minimum 30% sequence identity, minimum 30% of query aligned) to *A. lyrata*, *E. parvulum*, and RefSeq plants were recorded for all 27,016 genes. Cufflinks predicted genes were annotated with all of the above and the best hit in *A. thaliana*; this information was already provided by Phytozome v7.0 for annotated genes.

GO and GO slim terms were assigned to genes based on the best *A. thaliana* blast hit using GO annotation file AT_GO_GOSLIM.txt file retrieved from TAIR on May 10, 2012 (http://ftp.arabidopsis.org/home/tair/Ontologies/Gene_Ontology/). To account for the structure of GO, the Bioconductor package “AnnotationDbi” v1.16.11 was used to assign less specific GO terms to each gene.

Ribosomal RNA genes were identified on *E. salsugineum* scaffolds using the 18S, 5.8S, and 25S genes from *Sinapis alba* found in GenBank record X66325. These genes were found to be tandemly duplicated five times on scaffold 14 located between bases 7316449 and 7361125 (E-value ≤ 1^-50^, 96-98% sequence identity, 100% of query aligned).

*Eutrema* genes were identified as putatively associated with “Photosynthesis” (GO:0015979) and “Response to salt stress” (GO:009651) if their *Arabidopsis* orthologue is associated to these or their children terms.

#### Sequence polymorphism detection

Uniquely mapped reads were used as input for the mpileup function of SAMtools v0.1.18 [25,63] for initial identification of polymorphic loci. At each polymorphic position, the DepthOfCoverageWalker module from the GATK v1.3-19 [26] was used to filter mpileup calls and generate a list of high quality SNPs and InDels. Custom Perl scripts were used to exclude SNPs supported by fewer than 5 sequence reads or that were present at a frequency of less than 1% of informative reads. The initial set of polymorphisms detected included a small number of SNPs (944) for which there was evidence of more than two alleles. However, the additional alleles did not pass the rigorous filters described so these SNPs are included on the master list of two-allele SNPs (Additional file 3).

A subset of 101 Shandong-specific SNP-associated loci were investigated within the BGI genome release by BLASTing (E-value ≤ 10^-20^) a 301 bp fragment of cDNA against BGI scaffolds. Using the best BLAST hit (lowest E-value), custom Perl scripts determined whether the nucleotide located at position 151 (putative SNP) was identical to the JGI genome release or our Shandong transcriptome data.

#### Identification of differentially expressed genes

The number of reads unambiguously assigned to each gene was determined with the htseq-count script from HTSeq v0.5.3p3 using the intersection-nonempty overlap resolution mode (http://www-huber.embl.de/users/anders/HTSeq) and summarizing counts at the gene level. In the JGI annotation, the gene *PGR5* (At2g05620/Thhalv10002723m.g) is completely overlapped by the 3′ UTR of Thhalv10002628m.g (At2g05590) so no reads were counted for *PGR5* using the above parameters. However, inspection of our RNA-Seq alignments show that most reads mapped to this location are spliced reads so they can be unambiguously assigned to one of the two genes. Thus, read counts for *PGR5* were subsequently identified at the exon level with htseq-count and manually entered into Additional file 6 and Additional file 7.

The “DESeq” bioconductor package v1.6.1 [41] was used to normalize the read counts and call differentially expressed genes between each pair of conditions with default parameters. Genes were considered differentially expressed using a 0.05 false discovery rate (FDR) cutoff [64].

#### Gene ontology enrichment

GO enrichment was tested with GOseq v1.6.0 to reduce the biasing influence of long and/or abundantly expressed transcripts in RNAseq data [65] using the Wallenius approximation with gene lengths estimated as median mRNA lengths; the random sampling method with 400,000 iterations produced similar results (data not shown). GO slim categories were considered significantly over- or under-represented using a 0.05 FDR cutoff [64].

#### Multivariate analyses

Prior to statistical analyses performed with R v2.14.1 (R Development Core Team 2011), raw read counts were normalized by library size (per million uniquely mapped reads) and median mRNA length per gene (per kb of exon or UTR), shifted by a constant of 0.5, and log_10_ transformed. A heat map was generated for all 2,989 differentially regulated genes across all nine libraries using the heatmap.2 function in the “gplots” package; complete linkage clustering was performed on unscaled Euclidean distances between the log_10_ transformed expression values. Stability of sample clustering in the heat map was assessed using the R package “pvclust” [66] to calculate bootstrap probability and approximately unbiased *p*-values with 10,000 bootstrap replicates.

PCA was performed on the covariance matrix for all 27,016 genes across all nine libraries using a custom R script based on the “bpca” R package. Genes and libraries were factorized using the row metric preserving and squared root symmetric methods, respectively, prior to visualization.

### Validation of sequence polymorphisms by High Resolution Melting (HRM)

Using custom Perl scripts, a subset of SNPs and InDels were selected in which 1) all Yukon plants profiled shared an identical sequence variant in comparison to all the Shandong plants and 2) each polymorphism was supported by more than 20 Roche 454 sequence reads. Forty of these high-confidence polymorphisms, 33 SNPs and 7 InDels distinguishing the two *Eutrema* accessions, were selected at random for further HRM analysis. Primer-BLAST freeware (http://www.ncbi.nlm.nih.gov/tools/primer-blast/) was used to select primers generating a SNP-containing amplicon of no longer than 100 bp. Amplicon sequences were analyzed with MFOLD [67] (http://mfold.rna.albany.edu/?q=mfold/DNA-Folding-Form) and primer sets were rejected if free energies of any secondary structure were predicted to be more negative than −3.5 kcalmol^-1^[68].

*Eutrema* genomic DNA was extracted from approximately 100 mg of leaf tissue sourced from single plants. Frozen, powdered tissue was extracted first in an aqueous buffer (200 mM Tris–HCl pH 7.5, 250 mM NaCl, 25 mM Na-EDTA, 0.5% (w/v) SDS) then with phenol: chloroform: isoamyl alcohol (25:24:1). Genomic DNA was sequentially precipitated first with isopropanol, then with ethanol and DNA was resuspended in 0.1X TE buffer and frozen at −20°C until further analysis. DNA yield and quality were assessed on a Nanodrop 2000 spectrophotometer.

Quantitative PCR reactions were carried out on a Bio-Rad CFX96 Touch instrument using 50 ng genomic DNA and SsoFast EvaGreen Supermix (Bio-Rad). Amplicons from each genotype were obtained in five technical replicates. Melt curves were generated and analyzed with Precision Melt Analysis software (Bio-Rad) using default parameters.

### Availability of supporting data

The data discussed in this publication have been deposited in NCBI’s Gene Expression Omnibus (GEO) database [69] and are accessible through GEO Series accession GSE 49378 at (http://www. ncbi.nlm.nih.gov/geo/query/acc.cgi?acc = GSE49378).

## Competing interests

The authors declared that they have no competing interests.

## Authors’ contributions

MJC, RKC, GBG and EAW conceived and designed the experiments. MJC and RS performed the experiments: MJC, WLS, VC, SAD, NJP, GBG and EAW analyzed the data: MJC, PSS and EAW wrote the paper. All authors read and approved the final manuscript.

## Supplementary Material

Additional file 1: Figure S1 Cluster of rRNA genes that map to a single region of the genome. An average 42% of non-uniquely aligned reads across all nine libraries map to rRNA genes located between positions 7316449–7361125 on scaffold 14 of the JGI reference genome. The diagram illustrates a gene annotation (blue boxes) of the tandem array of rRNA genes on this scaffold as well as an alignment of all the sequence reads derived from rRNA (grey) that map to this location. Sequences were aligned with GMAP and visualized using the Integrative Genomics Viewer. **Figure S2**. Hierarchical clustering of genes differentially expressed in the nine *Eutrema* transcriptomes as well as a transcriptome of a cauline leaf from a cabinet-grown plant. Heat map of 2,989 differentially expressed genes generated using complete-linkage clustering of Euclidean distances between log-normalized expression values. Expression levels for genes in each cDNA library are represented as colors ranging from purple (little or no expression) to yellow (abundant expression). On the heat map, expression levels of DEGs in each library are also illustrated by black traces. Clustering at the level of individual libraries is represented by the dendrogram at the top of the heat map and clustering at the level of individual genes is presented at the left of the heat map. Click here for file

Additional file 2 ***Eutrema *genes for which the JGI reference genome has no annotation.** 665 genes supported by our RNA-Seq reads are present in the JGI *Eutrema* reference genome but are not annotated as genes. The location of each gene on JGI genome scaffolds is indicated. On this and subsequent tables, genes are annotated based on their best BLAST match to an *A. thaliana* and/or *A. lyrata, E. parvulum* gene or an entry in the RefSeq Plants database. The XLOC locus identifier indicates that the gene was not annotated by JGI. Click here for file

Additional file 3 **Two-allele *Eutrema *SNPs.** Rows provide summary statistics for each of the 74,550 identified SNPs. The polymorphism is represented by the reference allele in the JGI *Eutrema* genome (JGI REF allele = R) as well as the alternative allele contained in at least one plant studied (ALT allele = A). The number of sequence reads supporting each SNP call is included for every plant (coverage). The location of each SNP on the JGI genome scaffolds is indicated and 100 bp of flanking sequence at the locus is included to provide genomic context for each polymorphism. Genome sequence denoted by lower case text is highlighted as lower confidence sequence by JGI. Loci are ordered according to the non-overlapping groups presented in Table 5. 1 = unique to SC vs. JGI; 2 = unique to YC vs. JGI; 3 = unique to YF vs. JGI; 0 = remainder of the SNPs. Click here for file

Additional file 4 **Two-allele *Eutrema *InDels.** Each row provides summary statistics for an individual InDel, identified by its scaffold position on the JGI reference genome. The polymorphism is represented by the reference allele in the JGI *Eutrema* genome and the alternative allele contained in at least one plant studied. The zygosity of each InDel is indicated for every plant. 100 bp of flanking sequence at the locus is included to provide genomic context for each InDel. Click here for file

Additional file 5 ***Eutrema *genes with the highest density of SNPs.** Each row provides summary statistics for an individual *Eutrema* locus. SNP density was calculated as the number of SNPs present per unit 1 kb of sequence determined by median mRNA length. 40 of these genes were differentially regulated in our study and their expression patterns are also indicated according to the classification system presented in Table 5. n/a denotes those genes that were not differentially expressed. Click here for file

Additional file 6 **Expression levels of 27,016 genes in nine *Eutrema *plants.** Gene expression was calculated from the number of uniquely aligned reads at each locus. Values represent raw reads without normalization according to library size or gene length. Click here for file

Additional file 7 **Differentially expressed genes among cabinet- and field-grown *Eutrema *plants.** DESeq software was used to identify differentially expressed genes in three comparisons: SC vs. YC, YC vs. YF, SC vs. YF (one comparison per tab). All genes expressed at a significantly different level in at least one comparison (*p* <0.05 with FDR <0.05) are presented in each tab. The expression level in every plant and the fold-change in expression for a given comparison are also included. Click here for file

Additional file 8 **Transcripts whose loadings contribute the greatest to the negative PC3 position of YC scores in Figure **3**D.** The expression levels of genes for a cabinet-grown cauline leaf are also included. Click here for file

Additional file 9 **Expression of genes associated with photosynthesis in *Eutrema.*** Expression estimates for *Eutrema* genes orthologous to *Arabidopsis* genes participating in the “Photosynthesis” gene ontology are presented. Genes detected as differentially expressed in this study are indicated according to the patterns outlined in Table 5. Click here for file

Additional file 10 **Expression of genes associated with salt exposure in *Eutrema.*** Expression estimates for *Eutrema* genes orthologous to *Arabidopsis* genes participating in the “Response to salt” gene ontology are presented. Genes detected as differentially expressed in this study are indicated according to the patterns outlined in Table 5. Click here for file
