# Supplementary figures and images for: RNA-Seq effectively monitors gene expression in Eutrema salsugineum plants growing in an extreme natural habitat and in controlled growth cabinet conditions
Source: BMC Genomics. 2013 Aug 28;14:578. doi: 10.1186/1471-2164-14-578 (PMC3846481; doi:10.1186/1471-2164-14-578)

## Slide 1
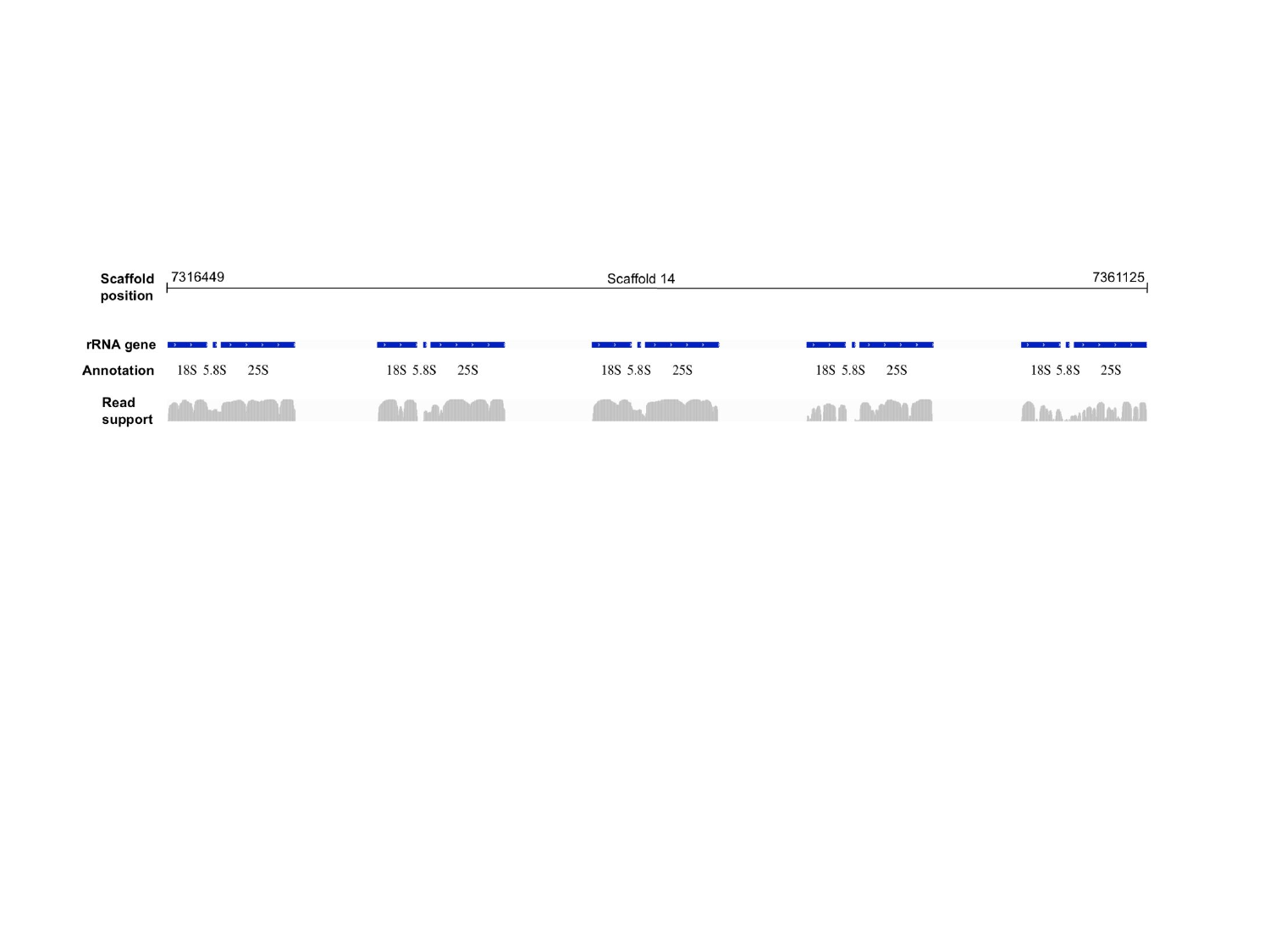

## Slide 2
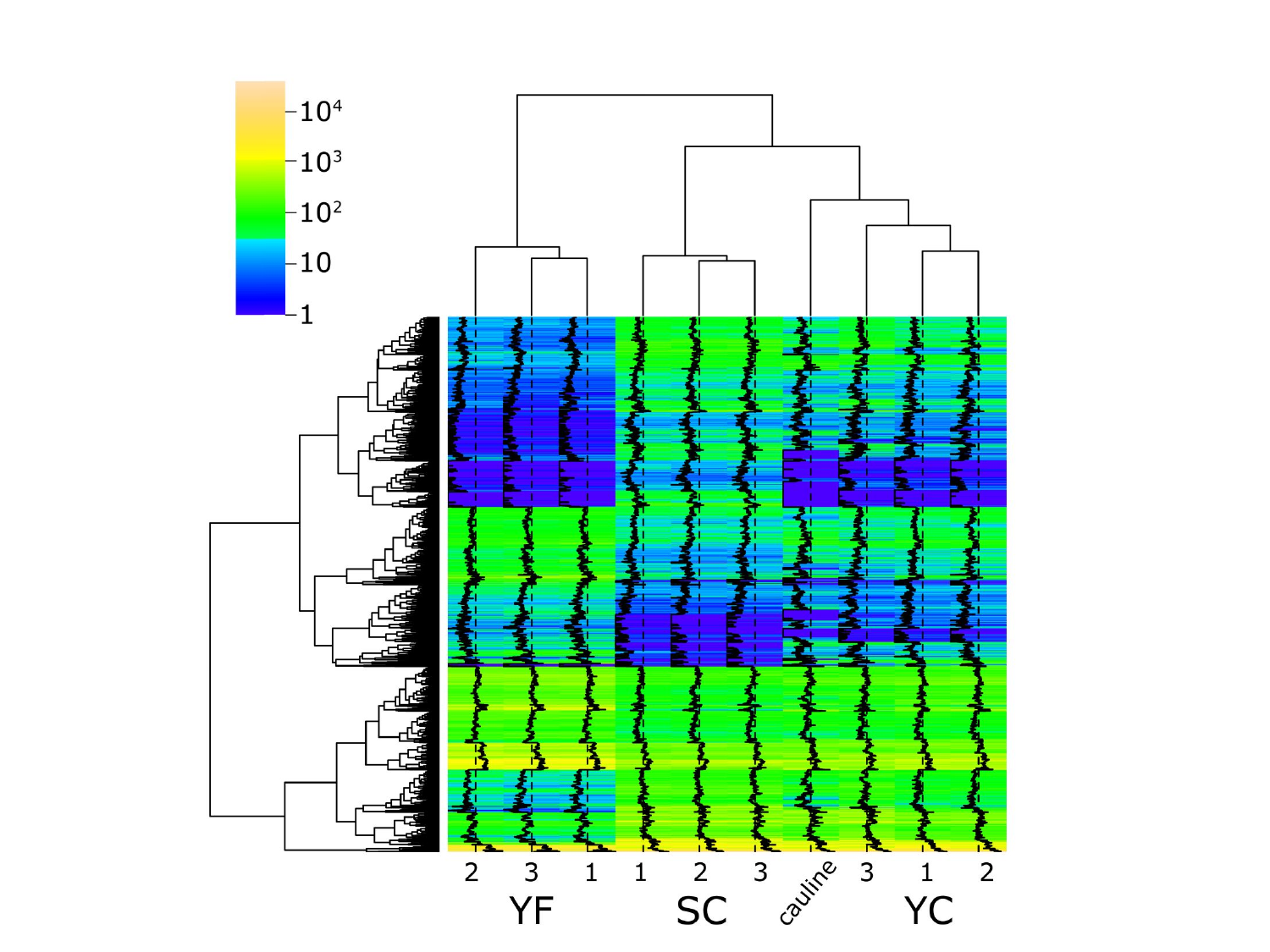

Supplement: Additional file 1: Figure S1 — Cluster of rRNA genes that map to a single region of the genome. An average 42% of non-uniquely aligned reads across all nine libraries map to rRNA genes located between positions 7316449–7361125 on scaffold 14 of the JGI reference genome. The diagram illustrates a gene annotation (blue boxes) of the tandem array of rRNA genes on this scaffold as well as an alignment of all the sequence reads derived from rRNA (grey) that map to this location. Sequences were aligned with GMAP and visualized using the Integrative Genomics Viewer. Figure S2. Hierarchical clustering of genes differentially expressed in the nine Eutrema transcriptomes as well as a transcriptome of a cauline leaf from a cabinet-grown plant. Heat map of 2,989 differentially expressed genes generated using complete-linkage clustering of Euclidean distances between log-normalized expression values. Expression levels for genes in each cDNA library are represented as colors ranging from purple (little or no expression) to yellow (abundant expression). On the heat map, expression levels of DEGs in each library are also illustrated by black traces. Clustering at the level of individual libraries is represented by the dendrogram at the top of the heat map and clustering at the level of individual genes is presented at the left of the heat map. [file 1471-2164-14-578-S1.pptx]
